# Genome-wide association studies of five free amino acid levels in rice

**DOI:** 10.3389/fpls.2022.1048860

**Published:** 2022-11-07

**Authors:** Liqiang He, Huixian Wang, Yao Sui, Yuanyuan Miao, Cheng Jin, Jie Luo

**Affiliations:** ^1^ College of Tropical Crops, Hainan University, Haikou, China; ^2^ Sanya Nanfan Research Institute of Hainan University, Hainan Yazhou Bay Seed Laboratory, Sanya, China

**Keywords:** rice, free amino acid level, genome-wide association study, quantitative trait locus, quantitative trait nucleotide-by-environment interactions

## Abstract

Rice (*Oryza sativa* L.) is one of the important staple foods for human consumption and livestock use. As a complex quality trait, free amino acid (FAA) content in rice is of nutritional importance. To dissect the genetic mechanism of FAA level, five amino acids’ (Val, Leu, Ile, Arg, and Trp) content and 4,325,832 high-quality SNPs of 448 rice accessions were used to conduct genome-wide association studies (GWAS) with nine different methods. Of these methods, one single-locus method (GEMMA), seven multi-locus methods (mrMLM, pLARmEB, FASTmrEMMA, pKWmEB, FASTmrMLM, ISIS EM-BLASSO, and FarmCPU), and the recent released 3VmrMLM were adopted for methodological comparison of quantitative trait nucleotide (QTN) detection and identification of stable quantitative trait nucleotide loci (QTLs). As a result, 987 QTNs were identified by eight multi-locus GWAS methods; FASTmrEMMA detected the most QTNs (245), followed by 3VmrMLM (160), and GEMMA detected the least QTNs (0). Among 88 stable QTLs identified by the above methods, 3VmrMLM has some advantages, such as the most common QTNs, the highest LOD score, and the highest proportion of all detected stable QTLs. Around these stable QTLs, candidate genes were found in the GO classification to be involved in the primary metabolic process, biosynthetic process, and catalytic activity, and shown in KEGG analysis to have participated in metabolic pathways, biosynthesis of amino acids, and tryptophan metabolism. Natural variations of candidate genes resulting in the content alteration of five FAAs were identified in this association panel. In addition, 95 QTN-by-environment interactions (QEIs) of five FAA levels were detected by 3VmrMLM only. GO classification showed that the candidate genes got involved in the primary metabolic process, transport, and catalytic activity. Candidate genes of QEIs played important roles in valine, leucine, and isoleucine degradation (QEI_09_03978551 and candidate gene *LOC_Os09g07830* in the Leu dataset), tryptophan metabolism (QEI_01_00617184 and candidate gene *LOC_Os01g02020* in the Trp dataset), and glutathione metabolism (QEI_12_09153839 and candidate gene *LOC_Os12g16200* in the Arg dataset) pathways through KEGG analysis. As an alternative of the multi-locus GWAS method, these findings suggested that the application of 3VmrMLM may provide new insights into better understanding FAA accumulation and facilitate the molecular breeding of rice with high FAA level.

## Introduction

Rice (*Oryza sativa* L.) is one of the most important crops worldwide and provides energy, amino acid, and dietary fiber for human consumption. In addition to the basic unit in protein biosynthesis, amino acids are involved in several cellular responses to affect physiological processes in plants, such as plant growth and development, intracellular pH control, production of metabolic energy or redox capacity, signal transduction, and response to abiotic and biotic stresses (Moe, 2013; Watanabe et al., 2013; Zeier, 2013; Fagard et al., 2014; Galili et al., 2014; Pratelli and Pilot, 2014: Hausler et al., 2014; Hildebrandt et al., 2015). Free amino acids (FAAs) not only play essential roles in plant growth, development, and responses to stress, but also serve as important nutrients for human health (Pathria and Ronai, 2021; Yang et al., 2022). Of all the amino acids, tryptophan (Trp), isoleucine (Ile), leucine (Leu), and valine (Val) are essential amino acids that are based on plants and cannot synthesize from external sources (Galili et al., 2016). In plants, branched-chain amino acids are important compounds in several aspects. Besides their function as building blocks of proteins, they get involved in the synthesis of a number of secondary products in plants and regulate plant growth by affecting the homeostasis of mineral elements in rice (Diebold et al., 2002; Jin et al., 2019). Arginine (Arg) is a semi-essential amino acid and involved in the regulation of various molecular pathways, which regulates key metabolic, immune, and neural signaling pathways in human cells (Patil et al., 2016). Branched-chain amino acids mainly including leucine, valine, and isoleucine generally participate in regulating protein synthesis, metabolism, food intake, and aging (Le Couteur et al., 2020). Arginine is a precursor of amino acids, polyamines, and nitric oxide (NO) for protein synthesis and is an important metabolite for many cells at the developmental stage (VanEtten et al., 1963; King and Gifford, 1997). Arginine is generally a major nitrogen storage form also in underground storage organs, roots of trees, and other plants (Bausenwein et al., 2001; Rennenberg et al., 2010). Tryptophan (Trp) is an aromatic amino acid that is synthesized through the shikimate/chorismate pathway. Notably, Trp is decarboxylated to tryptamine *in vivo*; subsequently, hydroxylase catalyzes the conversion of tryptamine to 5-hydroxytryptamine (5-HT). 5-HT is an important neurotransmitter associated with a range of human behavior problems such as personality and emotional disorders (Muller et al., 2016). Tryptophan provides the structural backbone for numerous plant secondary metabolites including the indoleamines, auxin [indole-3-acetic acid (IAA)], alkaloids, and benzoxazinoids (Erland and Saxena, 2019). Numerous loci with small effect underlying the natural variation of primary metabolites were found in previous studies (Rowe et al., 2008; Chan et al., 2010; Joseph et al., 2013; Fernie and Tohge, 2017). However, as one of primary metabolites, the genetic mechanism underlying these five FAA levels in rice is largely unknown, which is a limitation to the molecular breeding of rice with high-level FAAs.

Genome-wide association studies (GWAS) provide an insight into unraveling the genetic basis of complex traits in plants, especially for the trait controlled by small-effect genes (Zhu et al., 2008). Since the landmark GWAS of 107 Arabidopsis accessions (Atwell et al., 2010), GWAS of several agronomical traits in plants have been reported, which included starch content in wheat (Hao et al., 2020), flowering time and grain yield in rice (Yang et al., 2014; Liu et al., 2021), and seed protein and oil in soybean (Kim et al., 2021). With the technical progress and cost reduction of metabolomics, metabolite-based genome-wide association study (mGWAS) has been successfully applied in several functional genomics and metabolomics studies in plants (Luo, 2015; Fang et al., 2016; Fang and Luo, 2019).

Previous studies have proven the effectively controlled spurious association of widely adopted single-locus GWAS methods (Yu et al., 2006; Zhou and Stephens, 2012). However, the stringent Bonferroni correction is commonly used as the significant threshold of marker–trait associations (MTAs), which may result in the low power of polygenic loci detection in these methods (Zhang Y.M. et al., 2019). Thus, multi-locus GWAS methods have been proposed and identified quantitative trait nucleotide/locus (QTN/QTL) with small effect in a powerful manner (Segura et al., 2012). For instance, the improved statistical power and short computing time have been shown in the implementation of the FarmCPU method (Liu et al., 2016). The improvement of power and accuracy of the multi-locus GWAS method mrMLM have been reported (Wang et al., 2016). Additionally, a series of multi-locus models were proposed and released in R package mrMLM, which contained mrMLM (Wang et al., 2016), pLARmEB (Zhang et al., 2017), FASTmrEMMA (Wen et al., 2017), pKWmEB (Ren et al., 2017), FASTmrMLM (https://cran.r-project.org/web/packages/mrMLM/index.html), and ISIS EM-BLASSO (Tamba et al., 2017). However, the additive and dominance effects of trait-associated loci remain unclear. To address this issue, a new multi-locus GWAS method, 3VmrMLM, was proposed to estimate the genetic effects of three marker genotypes (AA, Aa, and aa) by controlling all the possibly polygenic backgrounds. Subsequently, these effects were further divided into additive and dominance effects for QTNs. Moreover, QTN-by-environment interactions (QEIs) were also able to be detected by 3VmrMLM for dissecting the genetic architecture of complex and multi-omics traits in GWAS (Li et al., 2022a).

To identify the QTLs associated with five FAAs levels, GWAS was performed on a genetic panel including 448 accessions with 4,325,832 SNPs from the rice core collection using nine statistical methods. Of these methods, one single-locus method, seven previous released multi-locus methods, and the recent proposed 3VmrMLM method were employed to determine the reliable approaches for main-effect QTLs and QEI detection of five FAA contents.

## Materials and methods

### Genetic panel for GWAS

A genetic panel of 448 rice accessions from our lab—a previously released core collection by Chen et al. (2014)—was used in Huazhong Agricultural University. It included 293 *indica* and 155 *japonica* accessions, of which 362 varieties are from Asia, 22 varieties are from America, 8 rice accessions are from Africa, 13 accessions are from Europe, 3 varieties are from Oceania and, 40 varieties have unknown geographical information.

### Metabolite profiling and sequencing

Two biological replicates of the 448 rice accessions grew in the normal rice growing season at two different blocks of Huazhong Agricultural University, Wuhan, China. For each replicate, randomly designed planting materials were used to harvest leaves at the five-leaf stage in liquid nitrogen of three different plants in each row of the field for metabolite extraction. Then, mix the material for biological replicate of each accession. The broad-sense heritability *H* ^2^ was calculated by using the data collected from different biological replicates at two different experimental bases of Huazhong Agricultural University. A scheduled multiple reaction monitoring (MRM) method with an MRM detection window of 80 s and a target scan time of 1.5 s were used to quantify the FAAs (Chen et al., 2013). Log_2_-transformed metabolite data were used for further analysis to improve normality.

To identify the genetic variation of 448 rice accessions, approximately 448 Gb high-quality genome sequences of these accessions were obtained from the Illumina HiSeq 2000 platform (Chen et al., 2014). Rice reference genome sequence MSU 6.1 (Nipponbare, version 6.1) and corresponding annotation were downloaded from Rice Genome Annotation Project (http://rice.uga.edu/index.shtml). Clean reads were mapped to the rice reference genome using BWA software (https://sourceforge.net/projects/bio-bwa/) with default settings. The mapping files were processed with SAMtools software (Li et al., 2009). HaplotypeCaller, CombineGVCFs and GenotypeGVCFs functions with default settings in GATK software (https://gatk.broadinstitute.org/hc/en-us) were used for SNP joint-calling and filter of the 448 accessions. Filtered high-quality SNPs (–maf 0.05 and –geno 0.1 in PLINK software, https://zzz.bwh.harvard.edu/plink/) were used for subsequent analysis.

### PCA and phylogenetic analysis

To summarize the genetic structure and variation of 448 rice accessions, principal component analysis (PCA) was conducted by PLINK software using the obtained high-quality SNPs. Furthermore, SNP-based phylogenetic analysis of all accessions was performed by MEGA-CC with a pairwise gap deletion method for 1,000 bootstrap replicates (Kumar et al., 2012).

### Population structure and linkage disequilibrium

ADMIXTURE software was employed to estimate the population stratification of all accessions (Alexander et al., 2009). To evaluate LD decay across the whole genome, the squared correlation coefficient (*r^2^*) between SNPs was computed and plotted using PopLDdecay software (Zhang C. et al., 2019).

### Genome-wide association study

GWAS were performed on the association panel containing 448 rice accessions with 4,325,832 high-quality SNPs. In total, nine models were implemented for GWAS, which included a single-locus model GEMMA (Zhou and Stephens, 2012) and eight multi-locus models, namely, FarmCPU (Liu et al., 2016), mrMLM (Wang et al., 2016), pLARmEB (Zhang et al., 2017), FASTmrEMMA (Wen et al., 2017), pKWmEB (Ren et al., 2017), FASTmrMLM (https://cran.r-project.org/web/packages/mrMLM/index.html), ISIS EM-BLASSO (Tamba et al., 2017), and 3VmrMLM (Li et al., 2022a). The R package mrMLM composed of six multi-locus methods mrMLM, pLARmEB, FASTmrEMMA, pKWmEB, FASTmrMLM, and ISIS EM-BLASSO was applied to test the marker and trait association. mrMLM parameter for six methods: Likelihood=“REML”, SearchRadius=20, CriLOD=3, SelectVariable=50, and Bootstrap=FALSE. These six methods in the mrMLM package were developed and released from the same research group that were referred to as “mrMLM series methods”. The LOD score ≥ 3 was used to detect the association signals of mrMLM series methods by default. The new released 3VmrMLM method, implemented by the IIIVmrMLM software (Li et al., 2022b), was used to detect main-effect quantitative trait nucleotide (QTN) and QTN by environment interaction (QEI). 3VmrMLM parameter for main-effect QTL: method=“Single_env”, SearchRadius=20, and svpal=0.01. 3VmrMLM parameter for QEI: method=“Multi_env”, SearchRadius=20, and svpal=0.01. The threshold of significant association of other methods was determined by a critical *p*-value at the 0.05 significant level subjected to Bonferroni correction (*p*-value = 1.16 × 10^−8^). All methods used in this study were implemented with default parameters. Manhattan and QQ plots were drawn using R CMplot, mrMLM, and 3VmrMLM packages with default settings.

### Analysis of candidate genes

Gene Ontology (GO) and Kyoto Encyclopedia of Genes and Genomes (KEGG) pathway annotation of candidate genes was analyzed by the Plant GeneSet Enrichment Analysis Toolkit (PlantGSEA) (Yi et al., 2013). The annotation of SNP effects on gene body was obtained from the RiceVarMap database (http://ricevarmap.ncpgr.cn/) and further used for haplotype and content analysis of potential candidate genes. Haplotype network was generated according to all information of a candidate gene from RiceVarMap database (http://ricevarmap.ncpgr.cn/). Temporal and spatial expression of potential candidate genes were assayed based on the expression data from electronic fluorescent pictograph Browser (ePlant) (http://bar.utoronto.ca/).

## Results

### FAA levels of rice genetic panel

**Figure 1:**
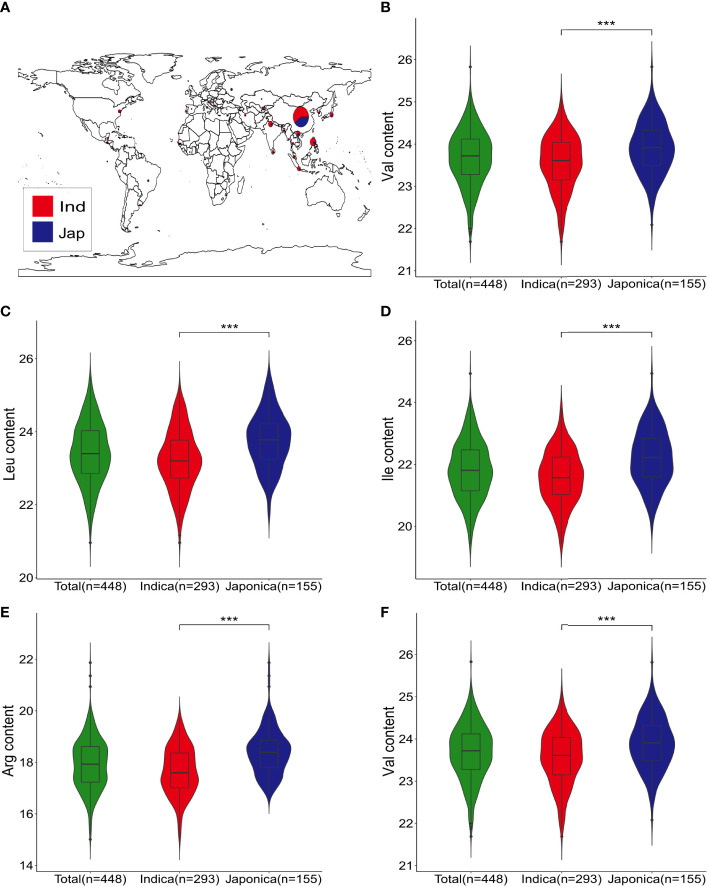
Geographic distribution and five FAA levels of genetic panel. **(A)** Geographic distribution of indica and japonica accessions in the genetic panel; indica accessions are indicated in red, and blue represents japionica accessions. **(B–F)** Violin plots of Val, Leu, Ile, Arg, and Trp contents for all, indica, and japonica accessions; *** indicate statistical significance at the 0.1% probability level

The five FAA levels (Val, Leu, Ile, Arg, and Trp) were quantified by LC-MS/MS to evaluate the phenotypic variation in 448 rice accessions. The CV of them were 45.03%, 58.83%, 71.25%, 92.30%, and 58.21%, respectively (**Table 1**). Furthermore, significant differences on five FAA levels were observed between *indica* and *japonica* accessions in this rice genetic panel (**Figure 1**). High correlation of five FAA contents was observed among them. For instance, the Val dataset was highly correlated with the Leu (*r* = 0.83) and Ile (*r* = 0.90) datasets, and the Leu dataset was highly correlated with the Ile (*r* = 0.93) dataset (**Supplementary Figure 1**). The skewness and kurtosis of five FAA levels were less than 1, which showed the nature of quantitative traits (**Supplementary Figure 1**; **Table 1**). The broad-sense heritability (*H* ^2^) for Val, Leu, Ile, Arg, and Trp ranged from 0.32 to 0.51 (**Table 1**). These indicated the natural variation of five amino acids present in this genetic panel.

**Table 1:** Descriptive statistics of five FAA content datasets.

Trait	Val	Leu	Ile	Arg	Trp
Number	448	448	448	448	448
Mean	23.68	23.41	21.80	17.91	22.20
Standard deviation	0.65	0.83	0.92	0.97	0.78
Variance	0.42	0.69	0.84	0.95	0.61
Mean squared error	0.03	0.04	0.04	0.05	0.04
Median	23.72	23.40	21.81	17.93	22.20
Trimmed	23.70	23.42	21.80	17.91	22.20
Median absolute deviation	0.61	0.87	0.98	1.02	0.82
Minimum	21.69	20.96	19.42	15.01	20.36
Maximum	25.83	25.51	24.94	21.87	24.47
Range	4.14	4.55	5.52	6.86	4.11
Skewness	−0.26	−0.07	0.05	0.10	0.04
Kurtosis	0.06	−0.31	−0.17	0.49	−0.27
^a^ Coefficient of variation (%)	45.03	58.83	71.25	92.30	58.21
Confidence interval of 0.95	0.06	0.08	0.09	0.09	0.07
*H* ^2^	0.32	0.51	0.46	0.38	0.43

a Calculated from the original dataset.

### Population structure and phylogenetic relationship of rice genetic panel

**Figure 2:**
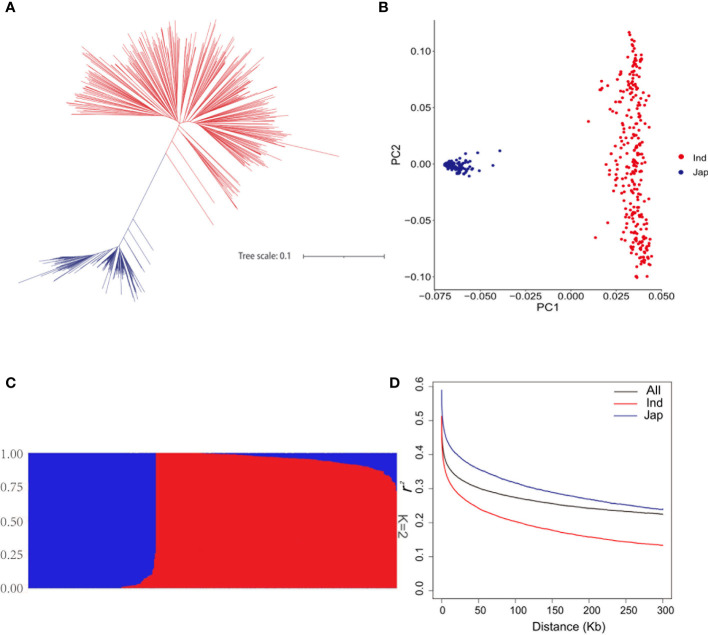
Population analyses of the genetic panel. **(A)** Phylogenetic tree of 448 rice accessions. **(B)** Principal component analysis of 448 rice accessions. **(C)** Population structure estimated by ADMIXTURE. **(D)** LD decay analysis of the genetic panel; LD decay of all 448 rice accessions, *indica* accessions, and *japonica* accessions is indicated in black, red, and blue, respectively.

To dissect the genetic basis underlying the natural variation of FAAs, the relationship assessment of rice genetic panel was based on 4,325,832 SNPs. According to the Neighbor-joining (NJ) phylogenetic tree, 448 rice accessions were mainly divided into two clades which contained 293 *indica* accessions and 155 *japonica* accessions, respectively (**Figure 2A**). Likewise, the classification of these accessions into two groups were observed in principal component analysis (PCA) (**Figure 2B**). Moreover, the population structure of rice genetic panel was identical with those obtained in NJ tree and PCA (**Figure 2C**). Linkage disequilibrium (LD) analysis showed that LD decayed fastest before 122 kb, and subsequently tended to be flat for the rice genetic panel (**Figure 2D**). Therefore, the 122- kb flanking region of each QTN was used for putative candidate gene prediction hereafter. Additionally, *indica* accessions showed the highest decay rate in **Figure 2D**.

### Identification of five FAA-associated QTLs

**Figure 3:**
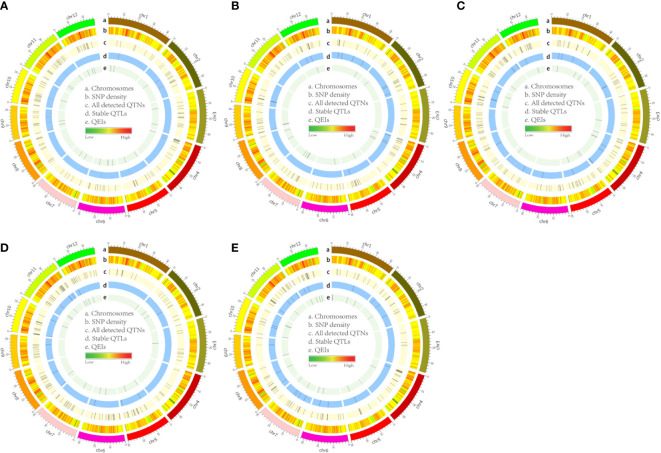
Circos map of QTLs and QEIs in rice genome identified from Val **(A)**, Leu **(B)**, Ile **(C)**, Arg **(D)**, and Trp **(E)** datasets. Track A: 12 rice chromosomes; Track B: heatmap of SNP density with bin sizes of 0.1 Mb; Track C: total unique QTNs detcted by all used methods; Track D: stable QTLs co-detected by no more than two methods; Track E: all detected QEIs by the 3VmrMLM method.

In this study, a total of 987 QTNs are identified using nine GWAS methods (a single-locus method, seven multi-locus methods, and the recently released 3VmrMLM method) for five FAA content datasets. Detected QTNs varied resulting from statistical methods (**Supplementary Table 1**). 3VmrMLM detected 160 QTNs and the largest number of common QTNs, while no QTN was detected by GEMMA. In addition, the largest number of QTNs were identified in the Trp dataset (214) by eight multi-locus GWAS methods (3VmrMLM, mrMLM, FASTmrEMMA, pLARmEB, FASTmrMLM, pKWmEB, ISIS EM-BLASSO, and FarmCPU), followed by the Val dataset (207), the Ile dataset (203), the Arg dataset (195), and the smallest number of detected QTNs in the Leu dataset (168) (**Figures 3A–E** and **Supplementary Figures 2A–E**; **Supplementary Table 1**). Six mrMLM series methods were compared together; FASTmrEMMA detected the most QTNs (245), followed by pLARmEB (160), mrMLM (151), FASTmrMLM (145), pKWmEB (77), and ISIS EM-BLASSO, which detected the least QTNs (25) (**Supplementary Figures 2A–E**; **Supplementary Table 1**). Different *R* ^2^ values of common QTNs across methods were observed, such as the *R* ^2^ value (%) of 3VmrMLM-detected QTNs that ranged from 0.78 to 6.95, while the *R* ^2^ value (%) of the mrMLM-detected QTN dataset was from 0.43 to 17.61. The average *R* ^2^ value (%) of ISIS EM-BLASSO-detected QTNs was the highest (2.93) among nine GWAS methods, whereas the average *R* ^2^ value (%) of the QTNs detected by FarmCPU was the lowest (0.24) (**Table 2**). Tag QTNs were selected and referred to as QTLs hereafter.

**Table 2:** Comparison of QTN/QTL identification for different GWAS methods.

Statistical method	No. of detected QTNs	No. of stable QTLs	Average *R* ^2^ (%)	*R* ^2^ range (%)	LOD range
3VmrMLM	160	83	1.99	0.78–6.95	3.04–46.29
FASTmrEMMA	245	29	1.01	0.01–8.93	3.01–24.01
FASTmrMLM	145	48	1.14	0.03–5.22	3.03–9.95
ISIS EM-BLASSO	25	9	2.93	0.98–6.89	3.01–10.65
mrMLM	151	19	2.54	0.43–17.61	3.06–21.49
pKWmEB	77	22	2.82	0.79–10.46	3.01–9.20
pLARmEB	160	34	1.46	0.01–14.39	3.02–14.80
FarmCPU	24	9	0.24	0.09–0.50	NA
GMMEA	0	0	NA	NA	NA

In addition, some common QTLs were detected in different FAA datasets. Intriguingly, QTL_01_10944343 (this QTL ID refers to QTL_Chromosome_Position) and QTL_05_19754561 were associated with Val and Ile datasets, respectively; QTL_01_23419417 was co-detected in the Leu and Ile datasets; QTL_02_24189963 was co-localized in the Leu and Trp datasets; QTL_09_16065720 was detected in the Arg and Trp datasets simultaneously; and QTL_10_17905052 was identified in the Ile and Arg datasets (**Supplementary Figure 3**). Among nine GWAS methods, most *p*-values of the 3VmrMLM-detected common QTLs were the lowest and most of their LOD scores were the highest correspondingly (**Table 2**; **Supplementary Table 1**; **Supplementary Figure 3**). These results indicated that the common QTLs detected by 3VmrMLM across traits were more significant than those detected by other eight GWAS methods.

### Stable FAA-associated QTLs and candidate genes

**Figure 4:**
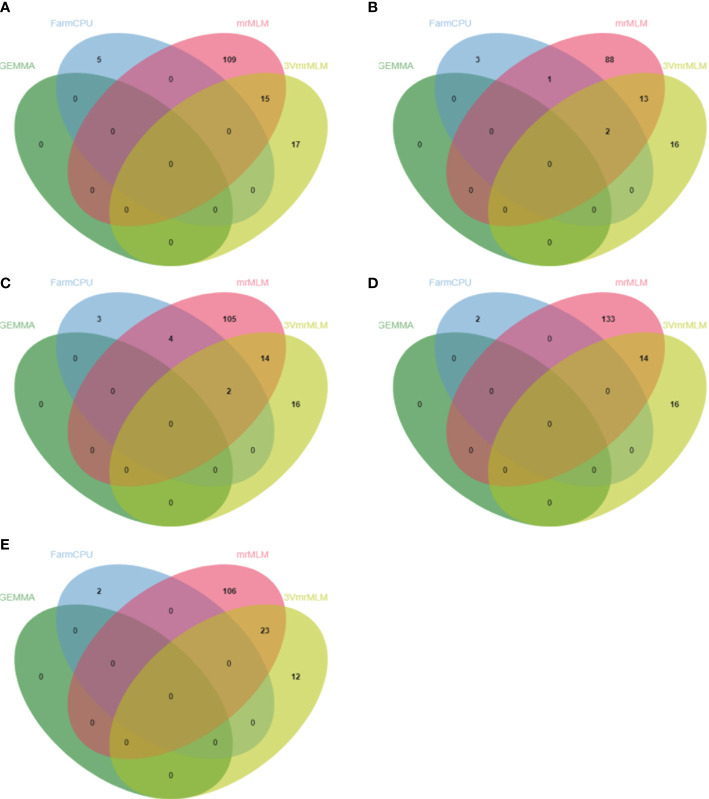
Venn diagrams of unique QTNs detected by different GWAS methods from Val **(A)**, Leu **(B)**, Ile **(C)**, Arg **(D)**, and Trp **(E)** datasets. mrMLM represents mrMLM series methods including mrMLM, FASTmrEMMA, pLARmEB, pKWmEB, ISIS EM-BLASSO, and FASTmrMLM.

**Figure 5:**
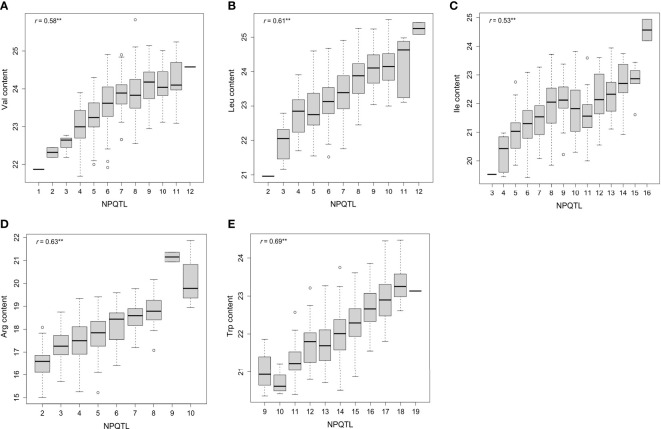
Box plots of the number of stable QTL with positive-effect alleles (NPQTL) in relation to Val, Leu, Ile, Arg, and Trp contents **(A–E)**. ** indicates statistical significance at the 1% probability level.

A QTL detected by no less than two methods of 3VmrMLM, mrMLM series methods (mrMLM, pLARmEB, FASTmrEMMA, pKWmEB, FASTmrMLM, and ISIS EM-BLASSO), FarmCPU, and GEMMA was defined as a stable QTL. A total of 88 stable QTLs were identified in five FAA datasets (**Supplementary Table 2**). Fifteen stable QTLs were detected in the Val dataset (**Figures 3A**, **4A**). In particular, QTL_01_10944343 was identified by seven GWAS methods (3VmrMLM, mrMLM, FASTmrMLM, FASTmrEMMA, pLARmEB, pKWmEB, and FarmCPU), and the QTL was also detected in Ile (**Supplementary Figure 3A**; **Supplementary Table 2**). For the Trp dataset, 23 stable QTLs were identified (**Figures 3E**, **4E**). Of these QTLs, QTL_09_16065720 was identified by six GWAS methods (3VmrMLM, FASTmrMLM, FASTmrEMMA, pLARmEB, pKWmEB, and ISIS EM-BLASSO), and it was detected in the Arg dataset simultaneously (**Supplementary Figure 3E**; **Supplementary Table 2**). Additionally, 16, 20, and 14 stable QTLs were detected in Leu, Ile, and Arg datasets (**Figures 3B–D**, **4B–D**). Significant correlations between NPQTL (the number of QTL with positive-effect or favorite alleles) and five FAA contents were observed in **Figures 5A–E** (*r* = 0.53–0.69). The highest correlation was shown in the Trp dataset (*r* = 0.69) (**Figure 5E**).

To understand the molecular basis controlling the five FAA levels, the biological function of candidate genes was investigated. According to functional annotations, these candidate genes were primarily categorized as protein, protein kinase, glycosyltransferase, and transcription factor (**Supplementary Table 3**). Furthermore, GO analysis showed that these genes were classified into 51 GO terms, such as the primary metabolic process, biosynthetic process, and catalytic activity (**Supplementary Figure 4**). Meanwhile, KEGG analysis of candidate genes showed that most of them were involved in metabolic pathways; biosynthesis of amino acids; glycine, serine, and threonine metabolism; and tryptophan metabolism (**Supplementary Figure 5**), for instance, biosynthesis of amino acids in five FAA datasets (**Supplementary Figures 5A–E**); glycine, serine, and threonine metabolism in the Leu dataset (**Supplementary Figure 5B**); and tryptophan metabolism in the Trp dataset (**Supplementary Figure 5E**).

**Figure 6:**
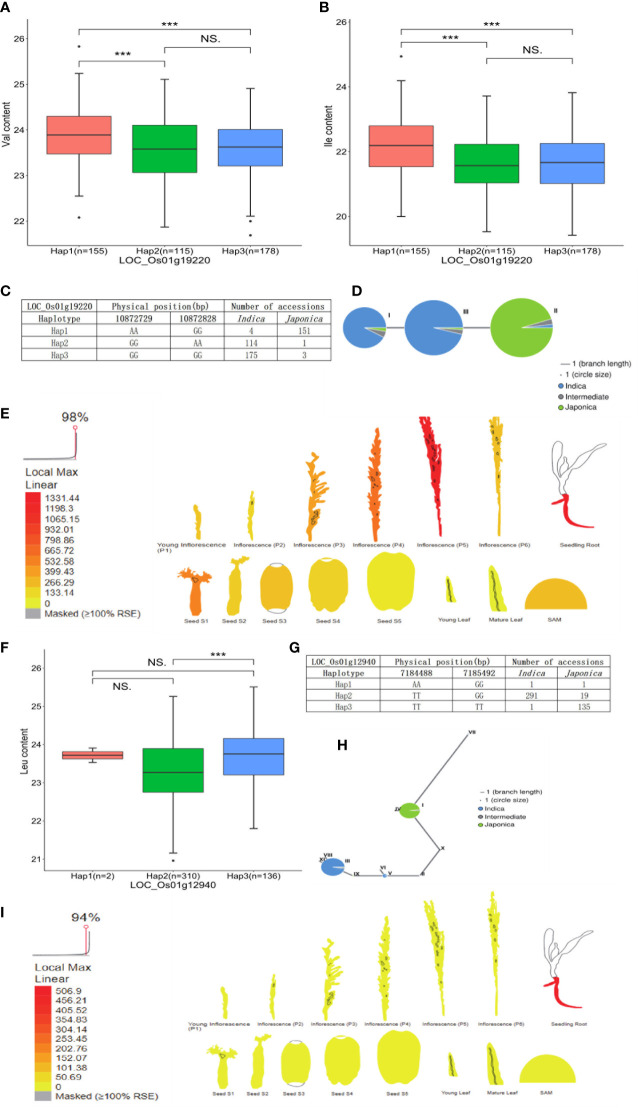
Analyses of Val and Ile level associated gene LOC_Os01g19220 and Leu level associated gene LOC_Os01g12940. **(A)** Significant tests between three haplotypes of LOC_Os01g19220 and Val contents. **(B)** Significant tests between three haplotypes of LOC_Os01g19220 and Ile contents. **(C)** Three haplotypes of LOC_Os01g19220 and their distribution in indica and japonica accessions. **(D)** Haplotype network of LOC_Os01g19220. **(E)** Expression profile of LOC_Os01g19220 based on ePlant transcriptome analysis in rice; expression strength coded by color from yellow (low) to red (high). **(F)** Significant tests between three haplotypes of LOC_Os01g12940 and Leu contents. **(G)** Three haplotypes of LOC_Os01g12940 and their distribution in indica and japonica accessions. **(H)** Haplotype network of LOC_Os01g12940. **(I)** Expression profile of LOC_Os01g12940 based on ePlant transcriptome analysis in rice, expression strength coded by color from yellow (low) to red (high). *** and NS indicate statistical significance at the 0.1% probability level and no significant difference, respectively.

**Figure 7:**
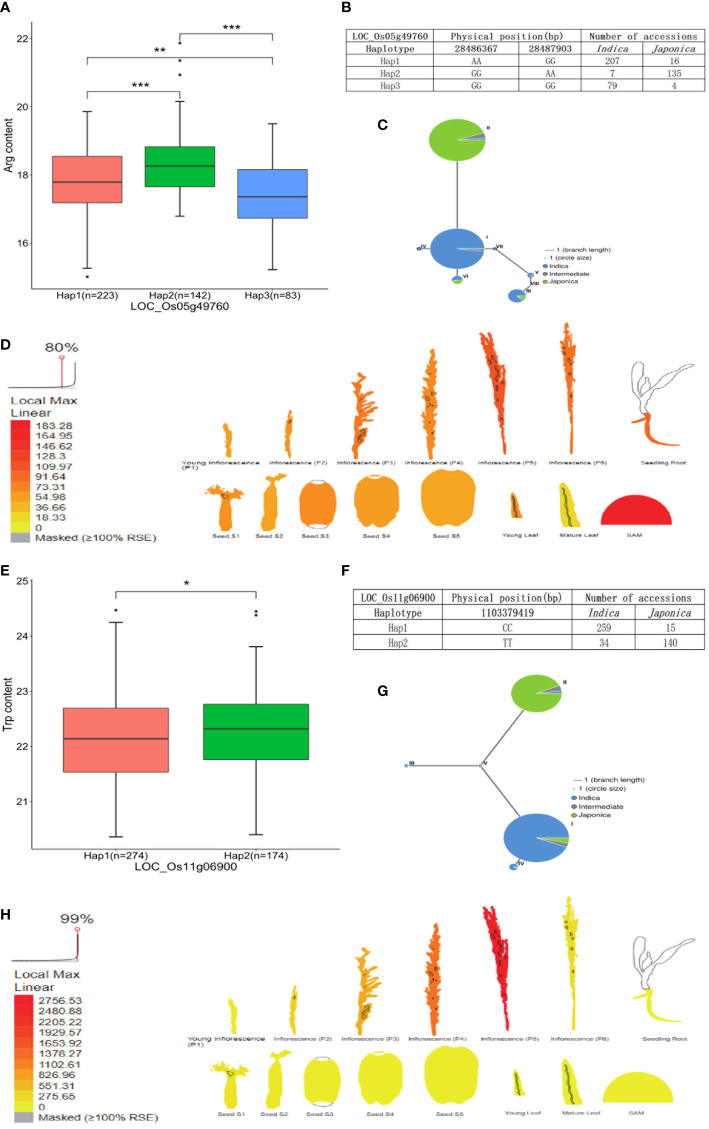
Analyses of Arg level associated gene *LOC_Os05g49760* and Trp level associated gene *LOC_Os11g06900*. **(A)** Significant tests between three haplotypes of *LOC_Os05g49760* and Arg contents. **(B)** Three haplotypes of *LOC_Os05g49760* and their distribution in *indica* and *japonica* accessions. **(C)** Haplotype network of *LOC_Os05g49760*. **(D)** Expression profile of *LOC_Os05g49760* based on ePlant transcriptome analysis in rice, expression strength coded by color from yellow (low) to red (high). **(E)** Significant tests between two haplotypes of *LOC_Os11g06900* and Trp contents. **(F)** Three haplotypes of *LOC_Os11g06900* and their distribution in *indica* and *japonica* accessions. **(G)** Haplotype network of *LOC_Os11g06900*. **(H)** Expression profile of *LOC_Os11g06900* based on ePlant transcriptome analysis in rice, expression strength coded by color from yellow (low) to red (high). *, **, and *** indicate statistical significance at the 5%, 1%, and 0.1% probability level, respectively.

The candidate gene *LOC_Os01g19220* encoding beta-D-xylosidase was identified in the Val and Ile datasets, which presented three types of alleles: Hap1 (AAGG) was concentrated in *japonica* accessions, while Hap2 (GGAA) and Hap3 (GGGG) were mainly concentrated in *indica* accessions, and the Val and Ile content of Hap1 was significantly different with the contents of Hap2 and Hap3. A lower Val and Ile content in Hap2 and Hap3 was observed than that in Hap1, which directly indicated the relatively high Val and Ile content present in *japonica* accessions compared with *indica* accessions (**Figures 6A–C**; **Supplementary Table 4**). Based on previous transcriptome and haplotype network analysis, *LOC_Os01g19220* was mainly expressed in seed (S1), inflorescence (P5), and seedling root. In the haplotype network, haplotype II of *LOC_Os01g19220* was mainly presented in *japonica* accessions; however, haplotypes I and III gathered in *indica* accessions (**Figures 6D, E**). Moreover, the gene *LOC_Os01g12940* encoding the phosphorylase domain containing protein detected in the Leu dataset had three types of allelic variation. Hap2 (TTGG) was concentrated in *indica* accessions, whereas Hap3 (TTTT) was concentrated in *japonica* accessions. A vast majority of *japonica* accessions with Hap3 showed significantly higher Leu level than *indica* accessions *with* Hap2 (**Figures 6F, G**; **Supplementary Table 4**). *LOC_Os01g12940* was highly expressed in seedling root. In the haplotype network, haplotype I of *LOC_Os01g12940* was concentrated in *japonica* accessions, while haplotypes III and V were concentrated in *indica* accessions (**Figures 6H,I**). In addition, the gene *LOC_Os05g49760* encoding the dehydrogenase is identified in the Arg dataset, which was involved in glutathione metabolism and had three types of allelic variation. Hap1 (AAGG) and Hap3 (GGGG) were enriched in *indica* accessions, and Hap2 (GGAA) was enriched in *japonica* accessions. Significant differences of Arg content were observed among accessions with Hap2, Hap1, and Hap3. Correspondingly, the Arg level of *japonica* accessions carrying Hap2 was higher than the *indica* accessions with Hap1 and Hap3 (**Figures 7A, B**; **Supplementary Table 4**). Relatively high abundance of *LOC_Os05g49760* was found in SAM (shoot apical meristem), young leaf, and inflorescence (P5). In the haplotype network, haplotype II was concentrated in *japonica* accessions, while haplotypes I and III gathered in *indica* accessions (**Figures 7C, D**). Moreover, the gene *LOC_Os11g06900* encoding amidase family protein detected in the Trp dataset had two alleles. Hap1 (CC) gathered in *indica* accessions, and Hap2 (TT) was mostly present in *japonica* accessions. Significant differences of Trp content were observed among accessions with Hap2 and Hap1. Subsequently, the Trp level of *japonica* accessions carrying Hap2 was higher than the *indica* accessions with Hap1 (**Figures 7E, F**; **Supplementary Table 4**). High expression of *LOC_Os11g06900* was observed in inflorescence (P5). In the haplotype network, haplotypes I, III, IV, and V of it gathered in *indica* accessions, whereas haplotype II was concentrated in *japonica* accessions (**Figures 7G, H**).

### QEI detection of five FAAs

In total, 95 QEIs of five FAAs were detected by 3VmrMLM (**Supplementary Table 5**). Of them, 23, 16, 16, 18, and 22 QEIs were identified in the Val, Leu, Ile, Arg, and Trp datasets (**Table 3**). However, no QEI was detected on some chromosomes in five FAA datasets (**Figure 3**; **Supplementary Figure 6**). For instance, no QEI on chromosomes 8 and 3 was found in the Val and Trp datasets, respectively (**Figures 3A, E**); none of the QEIs on chromosomes 3, 10, and 11 were detected in the Leu dataset (**Figure 3B**); no QEI located on chromosomes 6, 8, and 9 was identified in the Ile dataset (**Figure 3C**); and no QEI located on chromosomes 4 and 9 was identified in the Arg dataset (**Figure 3D**). Based on biological process, molecular function, and cellular component in GO analysis, candidate genes of these detected QEIs were classified into 47 GO terms, such as metabolic process, transferase activity, and transport (**Supplementary Figure 7**). Furthermore, KEGG pathway analysis showed that candidate genes were mainly involved in glutathione metabolism (QEI_12_09153839 and its candidate gene *LOC_Os12g16200* in the Arg dataset), valine leucine and isoleucine degradation (QEI_09_03978551 and its candidate gene *LOC_Os09g07830* in the Leu dataset), and tryptophan metabolism (QEI_01_00617184 and its candidate gene *LOC_Os01g02020* in the Trp dataset) (**Supplementary Figure 8** and **Supplementary Table 6**). In addition, cysteine and methionine metabolism in the Val dataset (**Supplementary Figure 8A**); tryptophan metabolism in the Trp dataset (**Supplementary Figure 8E**); and valine, leucine, and isoleucine degradation in the Leu dataset (**Supplementary Figure 8B**) are also shown in **Supplementary Figure 8**. According to ePlant analysis, high expression of *LOC_Os12g16200* encoding glutathione synthetase was observed in seedling root and mature leaf. *LOC_Os09g07830* encoding acetyl-CoA acetyltransferase was highly expressed in seedling root and SAM. Relatively high abundance of *LOC_Os01g02020* encoding acetyl-CoA acetyltransferase was found in young leaf and mature leaf.

**Table 3:** QTN-by-environment interactions (QEIs) detected from five FAA content datasets.

Trait	No. of detected QEIs	*R* ^2^ range (%)	LOD range	add*env1 range	add*env2 range
Val	23	0.33–2.42	5.09–35.28	−0.13–0.15	−0.15–0.13
Leu	16	0.57–2.41	5.07–21.31	−0.15–0.12	−0.12–0.15
Ile	16	0.46–2.94	4.83–29.92	−0.19–0.12	−0.12–0.19
Arg	18	0.34–1.22	6.16–21.15	−0.14–0.14	−0.14–0.14
Trp	22	0.36–2.60	4.63–34.53	−0.16–0.11	−0.11–0.16

## Discussion

### Methods comparison

Due to the difference of algorithm in different GWAS methods, the varied number of detected QTNs was observed accordingly. The FASTmrEMMA method detected the most QTNs (245), followed by 3VmrMLM (160), pLARmEB (160), mrMLM (151), FASTmrMLM (145), pKWmEB (77), ISIS EM-BLASSO (25), FarmCPU (24), and GEMMA, which detected the least QTNs (0) (**Supplementary Table 1**). Meanwhile, 3VmrMLM detected the largest number of common QTNs (**Figure 4**). Similar to the result obtained in this study, no QTN was identified in Xu et al. (2017) and Li et al. (2018) by GEMMA (Xu et al., 2017; Li et al., 2018). These were consistent with previous studies suggesting that multi-locus methods outperform single-locus methods on the statistical power of QTL detection, especially on the accuracy of QTN effect estimation and reduction of false-positive rate (Misra et al., 2017; Chang et al., 2018; Cui et al., 2018; Hou et al., 2018; Ma et al., 2018). The results of 3VmrMLM and mrMLM were compared as 3VmrMLM was a new three-variance component integrated with the mrMLM methodological framework. Most *p*-values of 3VmrMLM-detected QTNs were lower than those in mrMLM, and the LOD value of QTNs measured by 3VmrMLM was larger than the other eight methods (**Supplementary Figure 3**). These results indicated that the QTNs identified by 3VmrMLM were more significant than those identified by mrMLM. Additionally, the average *R* ^2^ value (%) of 3VmrMLM-detected QTNs was lower than that of mrMLM. The average *R* ^2^ value of ISIS EM-BLASSO (2.93) was the highest, followed by pKWmEB (2.82), mrMLM (2.54), 3VmrMLM (1.99), pLARmEB (1.46), FASTmrMLM (1.14), FASTmrEMMA (1.01), and FarmCPU (0.24) (**Table 2**). Notably, in this study, stable QTL_05_19754561 detected by 3VmrMLM/pLARmEB in the Val dataset, QTL_01_07646091 and QTL_07_08680072 detected by 3VmrMLM/mrMLM/pLARmEB/FarmCPU in the Ile dataset, QTL_11_22412156 detected by 3VmrMLM/pLARmEB in the Arg dataset, and QTL_01_23592545 detected by 3VmrMLM/FASTmrEMMA in the Trp dataset were reported in a previous study (Chen et al., 2014). Furthermore, QTN-0315484798 detected by 3VmrMLM only and QTN-0134428638 (~5.55 kb downstream of QTN-vg0134424130 detected by mrMLM in Ile dataset; QTN-0107646091 detected by FarmCPU/mrMLM in the Val/Trp dataset; QTN-0100694213, QTN-0727264573, and QTN-1203473916 detected by mrMLM/ISIS EM-BLASSO/pLARmEB in the Arg dataset; and QTN-0619805830 detected by ISIS EM-BLASSO and QTN-0805618520 detected by mrMLM in the Trp dataset were consistent with previous studies (Chen et al., 2014; Sun et al., 2020). Six QTLs (QTL_01_10944343, QTL_01_23419417, QTL_02_24189963, QTL_05_19754561, QTL_09_16065720, and QTL_10_17905052) were identified in more than one FAA dataset by no less than three methods (**Supplementary Figure 3**). Thus, the present complementarity of different methods suggested that the combined utilization of various single-locus and multi-locus GWAS methods may facilitate the identification of all potential QTLs with large and small effects in a powerful and robust manner, and the 3VmrMLM method may be used as an alternative for other multi-locus methods.

### Candidate genes for five FAA levels

A total of 88 stable QTLs were identified by no less than two methods. Genes co-localized in the 122-kb flanking region of stable QTL were identified for further analysis. Based on GO classification and KEGG pathway analysis, four potential candidate genes were found related to five FAA levels in rice, and the *Beta-glucosidase* gene (*LOC_Os01g19220*) involved in cyano amino acid metabolism (map00460) was a candidate gene of QTL_01_0944343 on chromosome 1, which was identified in both the Val and Ile datasets. According to KEGG pathway information, beta-glucosidase plays an important role in cyano amino acid metabolism, in which L-isoleucine and L-valine are required. The *Adenosylhomocysteine nucleosidase* gene (*LOC_Os01g12940*) associated with Leu content was identified in QTL_01_07089989 on chromosome 1 and involved in biosynthesis of amino acids (map01230) according to KEGG annotation. The *Isocitrate dehydrogenase* gene (*LOC_Os05g49760, IDH*) involved in glutathione metabolism (map00480) was detected in QTL_05_28394307 from the Arg dataset according to KEGG annotation. The *IDH* gene has been reported as a key enzyme in glutathione metabolism (Koh et al., 2004; Reitman et al., 2011; Tang et al., 2020). Glutathione is formed by the binding of γ-glutamate and cysteine *via* peptide bonds *via* the γ-glutamylcysteine synthetase (GSH1) and the binding of glycine catalyzed by glutathione synthetase (GSH2) (Noctor et al., 2012). As the essential precursor of glutathione, glutamate plays an important role in the biosynthetic and catabolism pathway of arginine. For instance, ornithine is synthesized from glutamate either in a cyclic or in a linear pathway and subsequently further converts to arginine; arginine catabolism begins with the degradation of arginine to ornithine, followed by the generation of glutamate through ornithine degradation (Winter et al., 2015; Majumdar et al., 2016). Genetic variation of *LOC_Os05g49760* resulted in the content alteration of Arg in this study (**Figure 7A**). The *Amidase* gene (*LOC_Os11g06900*) that participated in tryptophan metabolism (map00380) was a candidate gene of QTL_11_03441584 on chromosome 11, which was associated with Trp level in rice. In Arabidopsis, amidase catalyzes the conversion of indole-3-acetamide (IAM) to indole-3-acetic acid (IAA), which is an alternative terminal reaction step of IAA synthesis (Pollmann et al., 2009). IAA is the predominant auxin in plants, which can be synthesized from the Trp-dependent pathway. It has been confirmed that amidase promotes the synthesis of IAA, which is formed from tryptophan (Dharmasiri et al., 2005; Mockaitis and Estelle, 2008; Erland and Saxena, 2019). The natural variation of *LOC_Os11g06900* caused the content alteration of Trp in this study (**Figure 7E**). Moreover, *bZIP18*, *BCAT2*, and *BCAT4* genes have been validated to control the FAA levels in rice and other plant studies (Schuster et al., 2006; Angelovici et al., 2013; Sun et al., 2020). However, they were not found to be candidate genes of five FAA datasets in this study. Some transcript factors were co-localized with stable QTLs, which may contribute to the natural variation of FAA level in rice. Hence, the molecular mechanism of these candidate genes underlying the variation of FAA levels is warranted for further validation in the laboratory.

### Candidate gene prediction based on detected QEI

Compared with the other eight methods, 3VmrMLM is able to detect the QEI of five FAA levels. Based on the 95 detected QEIs, their predicted candidate genes were subjected to further functional analysis (**Supplementary Table 6**). According to KEGG annotation, the candidate gene *LOC_Os12g16200* of QEI_12_09153839 (this QEI ID refers to QEI_Chromosome_Position) encoding glutathione synthetase was identified in glutathione metabolism (map00480) in the Arg dataset. Glutathione synthetase (GSH) is an important enzyme to catalyze the formation of glutathione *via* the binding of γ-glutamate and cysteine (Noctor et al., 2012). Glutamate not only is an essential precursor for glutathione synthesis, but also participates in the biosynthetic and catabolism pathway of arginine (Noctor et al., 2012; Winter et al., 2015). *LOC_Os09g07830* of QEI_09_03978551 encoding acetyl-CoA acetyltransferase was identified in the Leu dataset, which was involved in valine leucine and isoleucine degradation (map00280) according to KEGG annotation. In the Trp dataset, *LOC_Os01g02020* gene harbored in QEI_01_00617184 encoding acetyl-CoA acetyltransferase was involved in tryptophan metabolism (map00380). These results suggested that a few QEIs may contribute to a small proportion of total variation on five FAA levels in rice.

### Breeding applications of FAA-associated QTLs

Significant correlations between NPQTL and five FAA contents were observed (*r* = 0.53–0.69), which indicated the additive effect of these QTLs, especially for the Trp dataset (*r* = 0.69) (**Figure 5**). It was observed that the highest levels of Arg were present in some rice accessions carrying nine QTLs with positive-effect or favorite alleles (PQTLs), such as C063 and W088. In addition, the Trp levels in accessions with 18 PQTLs (C119, etc.) were higher than those with 19 PQTLs (C197) (**Supplementary Table 7**). These suggested that the accessions carrying these PQTLs hold the potential in FAA biofortified rice breeding through the pyramiding of loci. This strategy has been successful in the improvement of FHB resistance in wheat (Buerstmayr et al., 2008). In five FAA datasets, FAA content in *japonica* accessions was generally higher than that in *indica* accessions (**Figures 1B–F**; **Supplementary Table 4**). This suggested that *japonica* accessions have more breeding potential than *indica* accessions in terms of these five FAA levels. These *japonica* accessions are good parents for genetic improvement of high FAA level by directly hybridizing with elite varieties. The average *R* ^2^ value of QTL detected in all five FAA datasets by 3VmrMLM was lower than that by mrMLM (**Table 2**). QTLs with a small effect have been successfully applied in genomic selection (GS) breeding for the improvement of disease resistance and yield in crops (Crossa et al., 2017; Wang et al., 2018; Xu et al., 2021). Hence, these relatively small-effect QTLs detected by 3VmrMLM might be applicable for genomic selection breeding in rice with high FAA levels; in particular, the 3VmrMLM method is beneficial for the QTL detection of an association mapping population consisting of heterozygous individuals (Li et al., 2022a).

## Conclusion

In this study, a total of 987 QTNs were detected in five FAA datasets by nine GWAS methods. The large number of detected QTNs demonstrated five FAA levels in rice were controlled by polygenes. 3VmrMLM has advantages in several aspects compared to other GWAS methods; 3VmrMLM detected the largest number of common QTNs, more significant on QTN detection, and relatively moderate *R* ^2^ values of QTLs were detected in multi-locus methods. The combined use of GWAS methods may facilitate the identification of all potential QTLs with large and small effects in a powerful and robust manner. Additionally, 15, 16, 20, 14, and 23 stable QTLs were detected in Val, Leu, Ile, Arg, and Trp datasets. Natural variations of the *LOC_Os01g19220* gene resulting in the content alteration of Val and Ile demonstrated that some potential candidate genes may play an important role in the crosslinking of different pathways. Of these QTLs, KEGG analysis of the candidate genes of five FAA-associated stable QTLs showed that they participated in biosynthesis of amino acids in five FAA datasets; glycine, serine, and threonine metabolism in the Leu dataset; and tryptophan metabolism in the Trp dataset. Moreover, 23, 16, 16, 18, and 22 QEIs were identified in the Val, Leu, Ile, Arg, and Trp datasets. KEGG pathway analysis showed that candidate genes were mainly involved in valine, leucine, and isoleucine degradation (QEI_09_03978551 and its candidate gene *LOC_Os09g07830* in the Leu dataset), tryptophan metabolism (QEI_01_00617184 and its candidate gene *LOC_Os01g02020* in the Trp dataset), and glutathione metabolism (QEI_12_09153839 and its candidate gene *LOC_Os12g16200* in the Arg dataset). To sum up, the combined utilization of 3VmrMLM with other GWAS methods will facilitate the mining of genes controlling complex traits and genomic selection breeding in rice.

## Data availability statement

The datasets presented in this study can be found in online repositories. The names of the repository/repositories and accession number(s) can be found in the article/**Supplementary Material**.

## Author contributions

LH conceived and designed this research project. YS, HW, and YM undertook the analysis of all available data. LH and HW contributed to resources and the writing of the original draft. JL and HL discussed the results, guided the entire study, participated in data analysis, and revised the manuscript. All authors contributed to the article and approved the submitted version.

## Funding

This study was supported by the Natural Science Foundation of Hainan Province (No. 321RC1148), the Key Research and Development Program of Hainan (No. ZDYF2020066), the “111” Project (No. D20024), the Hainan University Startup Fund KYQD (ZR) 1866 to J.L, and the Hainan University Startup Fund (RZ2100003217).

## Acknowledgments

We appreciate Wei Chen and other authors in Chen et al. (2014) for their great contribution to rice metabolic research field and public accessed data availability for reusage in this study.

## Conflict of interest

The authors declare that the research was conducted in the absence of any commercial or financial relationships that could be construed as a potential conflict of interest.

## Publisher’s note

All claims expressed in this article are solely those of the authors and do not necessarily represent those of their affiliated organizations, or those of the publisher, the editors and the reviewers. Any product that may be evaluated in this article, or claim that may be made by its manufacturer, is not guaranteed or endorsed by the publisher.

## Supplementary material

Supplementary Figure 1 Dot plots (lower triangle), histograms (diagonal) and Pearson correlations (upper triangle) between five FAAs datasets. Best curves are fitted in dot plots and histograms. *** indicates statistical significance at the 0.1% probability level probability level, and the size of the coefficient value is proportional to the strength of the correlation.

Supplementary Figure 2 Venn diagrams of unique QTNs detected by mrMLM series methods from Val **(A)**, Leu **(B)**, Ile **(C)**, Arg **(D)** and Trp **(E)**.

Supplementary Figure 3 Common QTNs detected in different FAA datasets by different methods. **(A)**: QTN-0110944343; **(B)**: QTN-0123419417; **(C)**: QTN-0224189963; **(D)**: QTN-0224189963; **(E)**: QTN-0519754561; **(F)**: QTN-0916065720; **(G)**: QTN-1017905052. The size of the circle is proportional to the significance level.

Supplementary Figure 4 GO classification of candidate genes harbored in stable QTLs in Val **(A)**, Leu **(B)**, Ile **(C)**, Arg **(D)**, Trp **(E)** datasets.

Supplementary Figure 5 KEGG pathway analysis of candidate genes harbored in stable QTLs in Val **(A)**, Leu **(B)**, Ile **(C)**, Arg **(D)** and Trp **(E)** datasets.

Supplementary Figure 6 Manhattan plots for five FAA levels detected QEIs by 3VmrMLM. QEIs in Val **(A)**, QEIs in Leu **(B)**, QEIs in Ile **(C)**, QEIs in Arg **(D)**, QEIs in Trp **(E)**. Black horizontal lines in the Manhattan plots represent the genome-wide significant threshold.

Supplementary Figure 7 GO classification of candidate genes harbored in QEIs in Val **(A)**, Leu **(B)**, Ile **(C)**, Arg **(D)** and Trp **(E)** datasets.

Supplementary Figure 8 KEGG pathway analysis of candidate genes harbored in QEIs in Val **(A)**, Leu **(B)**, Ile **(C)**, Arg **(D)** and Trp **(E)** datasets.

## References

[B1] Alexander D. H. Novembre J. Lange K. (2009). Fast model-based estimation of ancestry in unrelated individuals. Genome Res. 19, 1655–1664. doi: 10.1101/gr.094052.109 19648217 PMC2752134

[B2] Angelovici R. Lipka A. E. Deason N. Gonzalez-Jorge S. Lin H. Cepela J.. (2013). Genome-wide analysis of branched-chain amino acid levels in arabidopsis seeds. Plant Cell. 25, 4827–4843. doi: 10.1105/tpc.113.119370 24368787 PMC3903990

[B3] Atwell S. Huang Y. S. Vilhjalmsson B. J. Willems G. Horton M. Li Y.. (2010). Genome-wide association study of 107 phenotypes in arabidopsis thaliana inbred lines. Nature 465, 627–631. doi: 10.1038/nature08800 20336072 PMC3023908

[B4] Bausenwein U. Millard P. Thornton B. Raven J. A. (2001). Seasonal nitrogen storage and remobilization in the forb rumex acetosa. Funct. Ecol. 15, 370–377. doi: 10.1046/j.1365-2435.2001.00524.x

[B5] Buerstmayr H. Ban T. Anderson J. (2008). QTL mapping and marker assisted selection for fusarium head blight resistance in wheat. Cereal Res. Commun. 36, 1–3. doi: 10.1556/CRC.36.2008.Suppl.B.1

[B6] Chang F. Guo C. Sun F. Zhang J. Wang Z. Kong J.. (2018). Genome-wide association studies for dynamic plant height and number of nodes on the main stem in summer sowing soybeans. Front. Plant Sci. 9. doi: 10.3389/fpls.2018.01184 PMC6110304 30177936

[B7] Chan E. K. Rowe H. C. Hansen B. G. Kliebenstein D. J. (2010). The complex genetic architecture of the metabolome. PloS Genet. 6, e1001198. doi: 10.1371/journal.pgen.1001198 21079692 PMC2973833

[B8] Chen W. Gao Y. Xie W. Gong L. Lu K. Wang W.. (2014). Genome-wide association analyses provide genetic and biochemical insights into natural variation in rice metabolism. Nat. Genet. 46, 714–721. doi: 10.1038/ng.3007 24908251

[B9] Chen W. Gong L. Guo Z. Wang W. Zhang H. Liu X.. (2013). A novel integrated method for large-scale detection, identification, and quantification of widely targeted metabolites: application in the study of rice metabolomics. Mol. Plant 6, 1769–1780. doi: 10.1093/mp/sst0 23702596

[B10] Crossa J. Perez-Rodriguez P. Cuevas J. Montesinos-Lopez O. Jarquin D. de Los Campos G.. (2017). Genomic selection in plant breeding: Methods, models, and perspectives. Trends Plant Sci. 22, 961–975. doi: 10.1016/j.tplants.2017.08.011 28965742

[B11] Cui Y. Zhang F. Zhou Y. (2018). The application of multi-locus GWAS for the detection of salt-tolerance loci in rice. Front. Plant Sci. 9. doi: 10.3389/fpls.2018.01464 PMC6180169 30337936

[B12] Dharmasiri N. Dharmasiri S. Estelle M. (2005). The f-box protein TIR1 is an auxin receptor. Nature 435, 441–445. doi: 10.1038/nature03543 15917797

[B13] Diebold R. Schuster J. Daschner K. Binder S. (2002). The branched-chain amino acid transaminase gene family in arabidopsis encodes plastid and mitochondrial proteins. Plant Physiol. 129, 540–550. doi: 10.1104/pp.001602 12068099 PMC161671

[B14] Erland L. A. E. Saxena P. (2019). Auxin driven indoleamine biosynthesis and the role of tryptophan as an inductive signal in *Hypericum perforatum* (L.). PloS One 14. doi: 10.1371/journal.pone.0223878 PMC6797091 31622392

[B15] Fagard M. Launay A. Clement G. Courtial J. Dellagi A. Farjad M.. (2014). Nitrogen metabolism meets phytopathology. J. Exp. Bot. 65, 5643–5656. doi: 10.1093/jxb/eru323 25080088

[B16] Fang C. Luo J. (2019). Metabolic GWAS-based dissection of genetic bases underlying the diversity of plant metabolism. Plant J. 97, 91–100. doi: 10.1111/tpj.14097 30231195

[B17] Fang C. Zhang H. Wan J. Wu Y. Li K. Jin C.. (2016). Control of leaf senescence by an MeOH-jasmonates cascade that is epigenetically regulated by OsSRT1 in rice. Mol. Plant 9, 1366–1378. doi: 10.1016/j.molp.2016.07.007 27477683

[B18] Fernie A. R. Tohge T. (2017). The genetics of plant metabolism. Annu. Rev. Genet. 51, 287–310. doi: 10.1146/annurev-genet-120116-024640 28876980

[B19] Galili G. Amir R. Fernie A. R. (2016). The regulation of essential amino acid synthesis and accumulation in plants. Annu. Rev. Plant Biol. 67, 153–178. doi: 10.1146/annurev-arplant-043015-112213 26735064

[B20] Galili G. Avin-Wittenberg T. Angelovici R. Fernie A. R. (2014). The role of photosynthesis and amino acid metabolism in the energy status during seed development. Front. Plant Sci. 5. doi: 10.3389/fpls.2014.00447 PMC4153028 25232362

[B21] Hao C. Jiao C. Hou J. Li T. Liu H. Wang Y.. (2020). Resequencing of 145 landmark cultivars reveals asymmetric Sub-genome selection and strong founder genotype effects on wheat breeding in China. Mol. Plant 13, 1733–1751. doi: 10.1016/j.molp.2020.09.001 32896642

[B22] Hausler R. E. Ludewig F. Krueger S. (2014). Amino acids–a life between metabolism and signaling. Plant Sci. 229, 225–237. doi: 10.1016/j.plantsci.2014.09.011 25443849

[B23] Hildebrandt T. M. Nunes Nesi A. Araujo W. L. Braun H. P. (2015). Amino acid catabolism in plants. Mol. Plant 8, 1563–1579. doi: 10.1016/j.molp.2015.09.005 26384576

[B24] Hou S. Zhu G. Li Y. Li W. Fu J. Niu E.. (2018). Genome-wide association studies reveal genetic variation and candidate genes of drought stress related traits in cotton (*Gossypium hirsutum* l.). Front. Plant Sci. 9, 1276. doi: 10.3389/fpls.2018.01276 30233620 PMC6129771

[B25] Jin C. Sun Y. Shi Y. Zhang Y. Chen K. Li Y.. (2019). Branched-chain amino acids regulate plant growth by affecting the homeostasis of mineral elements in rice. Sci. China Life Sci. 62, 1107–1110. doi: 10.1007/s11427-019-9552-8 31165351

[B26] Joseph B. Corwin J. A. Li B. Atwell S. Kliebenstein D. J. (2013). Cytoplasmic genetic variation and extensive cytonuclear interactions influence natural variation in the metabolome. Elife 2, e00776. doi: 10.7554/eLife.00776 24150750 PMC3791467

[B27] Kim M. S. Lozano R. Kim J. H. Bae D. N. Kim S. T. Park J. H.. (2021). The patterns of deleterious mutations during the domestication of soybean. Nat. Commun. 12, 97. doi: 10.1038/s41467-020-20337-3 33397978 PMC7782591

[B28] King J. E. Gifford D. J. (1997). Amino acid utilization in seeds of loblolly pine during germination and early seedling growth (I. arginine and arginase activity). Plant Physiol. 113, 1125–1135. doi: 10.1104/pp.113.4.1125 12223664 PMC158235

[B29] Koh H. J. Lee S. M. Son B. G. Lee S. H. Ryoo Z. Y. Chang K. T.. (2004). Cytosolic NADP^+^-dependent isocitrate dehydrogenase plays a key role in lipid metabolism. J. Biol. Chem. 279, 39968–39974. doi: 10.1074/jbc.M402260200 15254034

[B30] Kumar S. Stecher G. Peterson D. Tamura K. (2012). MEGA-CC: computing core of molecular evolutionary genetics analysis program for automated and iterative data analysis. Bioinformatics 28, 2685–2686. doi: 10.1093/bioinformatics/bts507 22923298 PMC3467750

[B31] Le Couteur D. G. Solon-Biet S. M. Cogger V. C. Ribeiro R. de Cabo R. Raubenheimer D.. (2020). Branched chain amino acids, aging and age-related health. Ageing Res. Rev. 64, 101198. doi: 10.1016/j.arr.2020.101198 33132154

[B32] Li H. Handsaker B. Wysoker A. Fennell T. Ruan J. Homer N.. (2009). The sequence Alignment/Map format and SAMtools. Bioinformatics 25, 2078–2079. doi: 10.1093/bioinformatics/btp352 19505943 PMC2723002

[B33] Li J. Tang W. Zhang Y. W. Chen K. N. Wang C. Liu Y.. (2018). Genome-wide association studies for five forage quality-related traits in sorghum (Sorghum bicolor l.). Front. Plant Sci. 9. doi: 10.3389/fpls.2018.01146 PMC6111974 30186292

[B34] Liu X. Huang M. Fan B. Buckler E. S. Zhang Z. (2016). Iterative usage of fixed and random effect models for powerful and efficient genome-wide association studies. PloS Genet. 12, e1005767. doi: 10.1371/journal.pgen.1005767 26828793 PMC4734661

[B35] Liu C. Tu Y. Liao S. Fu X. Lian X. He Y.. (2021). Genome-wide association study of flowering time reveals complex genetic heterogeneity and epistatic interactions in rice. Gene 770, 145353. doi: 10.1016/j.gene.2020.145353 33333227

[B36] Li M. Zhang Y. W. Xiang Y. Liu M. H. Zhang Y. M. (2022a). IIIVmrMLM: The r and c++ tools associated with 3VmrMLM, a comprehensive GWAS method for dissecting quantitative traits. Mol. Plant 15, 1251–1253. doi: 10.1016/j.molp.2022.06.002 35684963

[B37] Li M. Zhang Y. W. Zhang Z. C. Xiang Y. Liu M. H. Zhou Y. H.. (2022b). A compressed variance component mixed model for detecting QTNs and QTN-by-environment and QTN-by-QTN interactions in genome-wide association studies. Mol. Plant 15, 630–650. doi: 10.1016/j.molp.2022.02.012 35202864

[B38] Luo J. (2015). Metabolite-based genome-wide association studies in plants. Curr. Opin. Plant Biol. 24, 31–38. doi: 10.1016/j.pbi.2015.01.006 25637954

[B39] Majumdar R. Barchi B. Turlapati S. A. Gagne M. Minocha R. Long S.. (2016). Glutamate, ornithine, arginine, proline, and polyamine metabolic interactions: The pathway is regulated at the post-transcriptional level. Front. Plant Sci. 7. doi: 10.3389/fpls.2016.00078 PMC4754450 26909083

[B40] Ma L. Liu M. Yan Y. Qing C. Zhang X. Zhang Y.. (2018). Genetic dissection of maize embryonic callus regenerative capacity using multi-locus genome-wide association studies. Front. Plant Sci. 9. doi: 10.3389/fpls.2018.00561 PMC5933171 29755499

[B41] Misra G. Badoni S. Anacleto R. Graner A. Alexandrov N. Sreenivasulu N. (2017). Whole genome sequencing-based association study to unravel genetic architecture of cooked grain width and length traits in rice. Sci. Rep. 7, 12478. doi: 10.1038/s41598-017-12778-6 28963534 PMC5622062

[B42] Mockaitis K. Estelle M. (2008). Auxin receptors and plant development: a new signaling paradigm. Annu. Rev. Cell Dev. Biol. 24, 55–80. doi: 10.1146/annurev.cellbio.23.090506.123214 18631113

[B43] Moe L. A. (2013). Amino acids in the rhizosphere: from plants to microbes. Am. J. Bot. 100, 1692–1705. doi: 10.3732/ajb.1300033 23956051

[B44] Muller C. L. Anacker A. M. J. Veenstra-VanderWeele J. (2016). The serotonin system in autism spectrum disorder: From biomarker to animal models. Neuroscience 321, 24–41. doi: 10.1016/j.neuroscience.2015.11.010 26577932 PMC4824539

[B45] Noctor G. Mhamdi A. Chaouch S. Han Y. Neukermans J. Marquez-Garcia B.. (2012). Glutathione in plants: an integrated overview. Plant Cell Environ. 35, 454–484. doi: 10.1111/j.1365-3040.2011.02400.x 21777251

[B46] Pathria G. Ronai Z. A. (2021). Harnessing the Co-vulnerabilities of amino acid-restricted cancers. Cell Metab. 33, 9–20. doi: 10.1016/j.cmet.2020.12.009 33406406 PMC7837405

[B47] Patil M. D. Bhaumik J. Babykutty S. Banerjee U. C. Fukumura D. (2016). Arginine dependence of tumor cells: targeting a chink in cancer’s armor. Oncogene 35, 4957–4972. doi: 10.1038/onc.2016.37 27109103 PMC5457742

[B48] Pollmann S. Duchting P. Weiler E. W. (2009). Tryptophan-dependent indole-3-acetic acid biosynthesis by ‘IAA-synthase’ proceeds *via* indole-3-acetamide. Phytochemistry 70, 523–531. doi: 10.1016/j.phytochem.2009.01.021 19268331

[B49] Pratelli R. Pilot G. (2014). Regulation of amino acid metabolic enzymes and transporters in plants. J. Exp. Bot. 65, 5535–5556. doi: 10.1093/jxb/eru320 25114014

[B50] Reitman Z. J. Jin G. Karoly E. D. Spasojevic I. Yang J. Kinzler K. W.. (2011). Profiling the effects of isocitrate dehydrogenase 1 and 2 mutations on the cellular metabolome. Proc. Natl. Acad. Sci. U. S. A. 108, 3270–3275. doi: 10.1073/pnas.1019393108 21289278 PMC3044380

[B51] Rennenberg H. W ildhagen H. Ehlting B. (2010). Nitrogen nutrition of poplar trees. Plant Biol. (Stuttg.) 12, 275–291. doi: 10.1111/j.1438-8677.2009.00309.x 20398235

[B52] Ren W. L. Wen Y. J. Dunwell J. M. Zhang Y. M. (2017). pKWmEB: integration of kruskal-Wallis test with empirical bayes under polygenic background control for multi-locus genome-wide association study. Heredity. (Edinb). 120, 208–218. doi: 10.1038/s41437-017-0007-4 29234158 PMC5836593

[B53] Rowe H. C. Hansen B. G. Halkier B. A. Kliebenstein D. J. (2008). Biochemical networks and epistasis shape the *Arabidopsis thaliana* metabolome. Plant Cell. 20, 1199–1216. doi: 10.1105/tpc.108.058131 18515501 PMC2438456

[B54] Schuster J. Knill T. Reichelt M. Gershenzon J. Binder S. (2006). Branched-chain aminotransferase4 is part of the chain elongation pathway in the biosynthesis of methionine-derived glucosinolates in *Arabidopsis* . Plant Cell. 18, 2664–2679. doi: 10.1105/tpc.105.039339 17056707 PMC1626624

[B55] Segura V. Vilhjalmsson B. J. Platt A. Korte A. Seren U. Long Q.. (2012). An efficient multi-locus mixed-model approach for genome-wide association studies in structured populations. Nat. Genet. 44, 825–830. doi: 10.1038/ng.2314 22706313 PMC3386481

[B56] Sun Y. Shi Y. Liu G. Yao F. Zhang Y. Yang C.. (2020). Natural variation in the *OsbZIP18* promoter contributes to branched-chain amino acid levels in rice. New Phytol. 228, 1548–1558. doi: 10.1111/nph.16800 32654152

[B57] Tamba C. L. Ni Y. L. Zhang Y. M. (2017). Iterative sure independence screening EM-Bayesian LASSO algorithm for multi-locus genome-wide association studies. PloS Comput. Biol. 13, e1005357. doi: 10.1371/journal.pcbi.1005357 28141824 PMC5308866

[B58] Tang X. Fu X. Liu Y. Yu D. Cai S. J. Yang C. (2020). Blockade of glutathione metabolism in IDH1-mutated glioma. Mol. Cancer Ther. 19, 221–230. doi: 10.1158/1535-7163.MCT-19-0103 31548295 PMC6946871

[B59] VanEtten C. H. Wolff I. A. Jones Q. Miller R. W. (1963). Amino acid composition of seeds from 200 angiospermous plant species. J. Agric. Food Chem. 11, 399–410. doi: 10.1021/jf60129a016

[B60] Wang S. B. Feng J. Y. Ren W. L. Huang B. Zhou L. Wen Y. J.. (2016). Improving power and accuracy of genome-wide association studies *via* a multi-locus mixed linear model methodology. Sci. Rep. 6, 19444. doi: 10.1038/srep19444 26787347 PMC4726296

[B61] Wang X. Xu Y. Hu Z. Xu C. (2018). Genomic selection methods for crop improvement: Current status and prospects. Crop J. 6, 330–340. doi: 10.1016/j.cj.2018.03.001

[B62] Watanabe M. Balazadeh S. Tohge T. Erban A. Giavalisco P. Kopka J.. (2013). Comprehensive dissection of spatiotemporal metabolic shifts in primary, secondary, and lipid metabolism during developmental senescence in arabidopsis. Plant Physiol. 162, 1290–1310. doi: 10.1104/pp.113.217380 23696093 PMC3707545

[B63] Wen Y. J. Zhang H. Ni Y. L. Huang B. Zhang J. Feng J. Y.. (2017). Methodological implementation of mixed linear models in multi-locus genome-wide association studies. Brief Bioinform. 19, 700–712. doi: 10.1093/bib/bbw145 PMC6054291 28158525

[B64] Winter G. Todd C. D. Trovato M. Forlani G. Funck D. (2015). Physiological implications of arginine metabolism in plants. Front. Plant Sci. 6. doi: 10.3389/fpls.2015.00534 PMC4520006 26284079

[B65] Xu Y. Ma K. Zhao Y. Wang X. Zhou K. Yu G.. (2021). Genomic selection: A breakthrough technology in rice breeding. Crop J. 9, 669–677. doi: 10.1016/j.cj.2021.03.008

[B66] Xu Y. Xu C. Xu S. (2017). Prediction and association mapping of agronomic traits in maize using multiple omic data. Heredity. (Edinb). 119, 174–184. doi: 10.1038/hdy.2017.27 28590463 PMC5564377

[B67] Yang W. Guo Z. Huang C. Duan L. Chen G. Jiang N.. (2014). Combining high-throughput phenotyping and genome-wide association studies to reveal natural genetic variation in rice. Nat. Commun. 5, 5087. doi: 10.1038/ncomms6087 25295980 PMC4214417

[B68] Yang J. Zhou Y. Jiang Y. (2022). Amino acids in rice grains and their regulation by polyamines and phytohormones. Plants (Basel) 11, 1581. doi: 10.3390/plants11121581 35736731 PMC9228293

[B69] Yi X. Du Z. Su Z. (2013). PlantGSEA: a gene set enrichment analysis toolkit for plant community. Nucleic Acids Res. 41, W98–103. doi: 10.1093/nar/gkt281 23632162 PMC3692080

[B70] Yu J. Pressoir G. Briggs W. H. Vroh Bi I. Yamasaki M. Doebley J. F.. (2006). A unified mixed-model method for association mapping that accounts for multiple levels of relatedness. Nat. Genet. 38, 203–208. doi: 10.1038/ng1702 16380716

[B71] Zeier J. (2013). New insights into the regulation of plant immunity by amino acid metabolic pathways. Plant Cell Environment. 36, 2085–2103. doi: 10.1111/pce.12122 23611692

[B72] Zhang C. Dong S.-S. Xu J.-Y. He W.-M. Yang T.-L. Schwartz R. (2019). PopLDdecay: a fast and effective tool for linkage disequilibrium decay analysis based on variant call format files. Bioinformatics 35, 1786–1788. doi: 10.1093/bioinformatics/bty875 30321304

[B73] Zhang J. Feng J. Y. Ni Y. L. Wen Y. J. Niu Y. Tamba C. L.. (2017). pLARmEB: integration of least angle regression with empirical bayes for multilocus genome-wide association studies. Heredity. (Edinb). 118, 517–524. doi: 10.1038/hdy.2017.8 28295030 PMC5436030

[B74] Zhang Y. M. Jia Z. Dunwell J. M. (2019). Editorial: The applications of new multi-locus GWAS methodologies in the genetic dissection of complex traits. Front. Plant Sci. 10. doi: 10.3389/fpls.2019.00100 PMC6378272 30804969

[B75] Zhou X. Stephens M. (2012). Genome-wide efficient mixed-model analysis for association studies. Nat. Genet. 44, 821–824. doi: 10.1038/ng.2310 22706312 PMC3386377

[B76] Zhu C. Gore M. Buckler E. S. Yu J. (2008). Status and prospects of association mapping in plants. Plant Genome. 1, 5–20. doi: 10.3835/plantgenome2008.02.0089

