# Supplementary material for: Genome-wide association studies of five free amino acid levels in rice
Source: Front Plant Sci. 2022 Nov 7;13:1048860. doi: 10.3389/fpls.2022.1048860 (PMC9676653; doi:10.3389/fpls.2022.1048860)
Supplement: Supplementary Figure 1 — Dot plots (lower triangle), histograms (diagonal) and Pearson correlations (upper triangle) between five FAAs datasets. Best curves are fitted in dot plots and histograms. *** indicates statistical significance at the 0.1% probability level probability level, and the size of the coefficient value is proportional to the strength of the correlation. [file Image_1.pdf]

Supplementary Figure 1

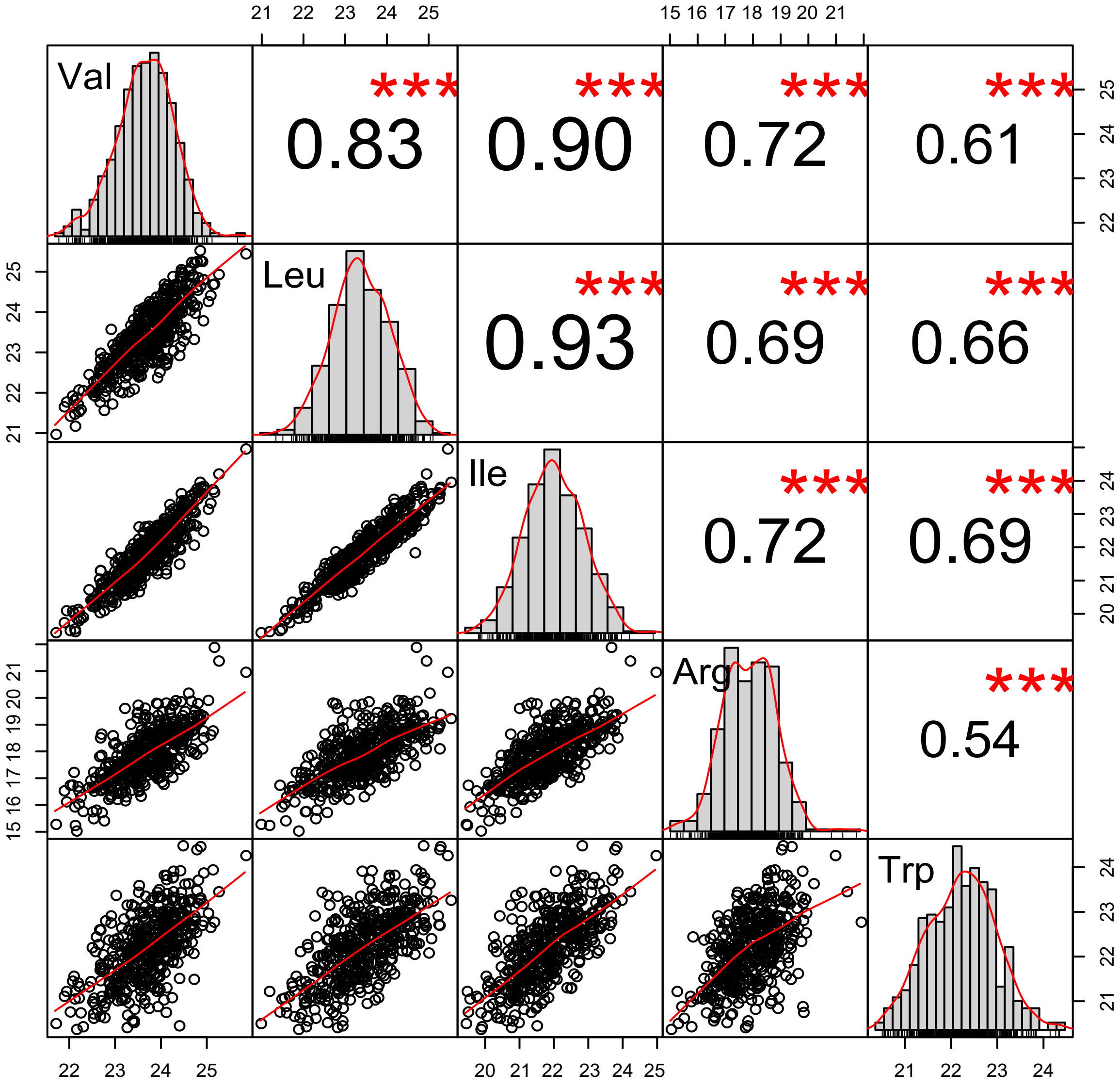

Supplementary Figure 2

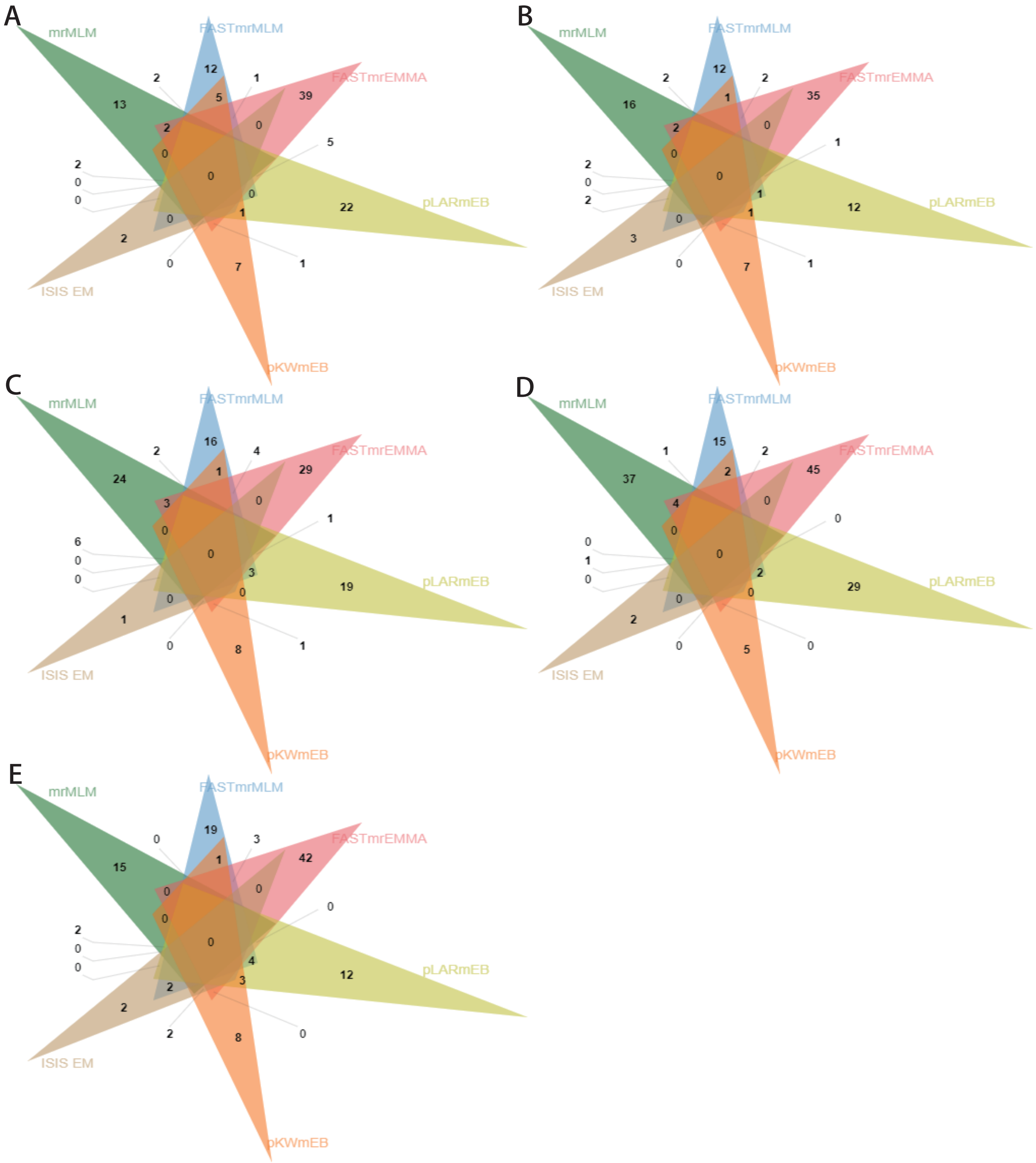

Supplementary Figure 3

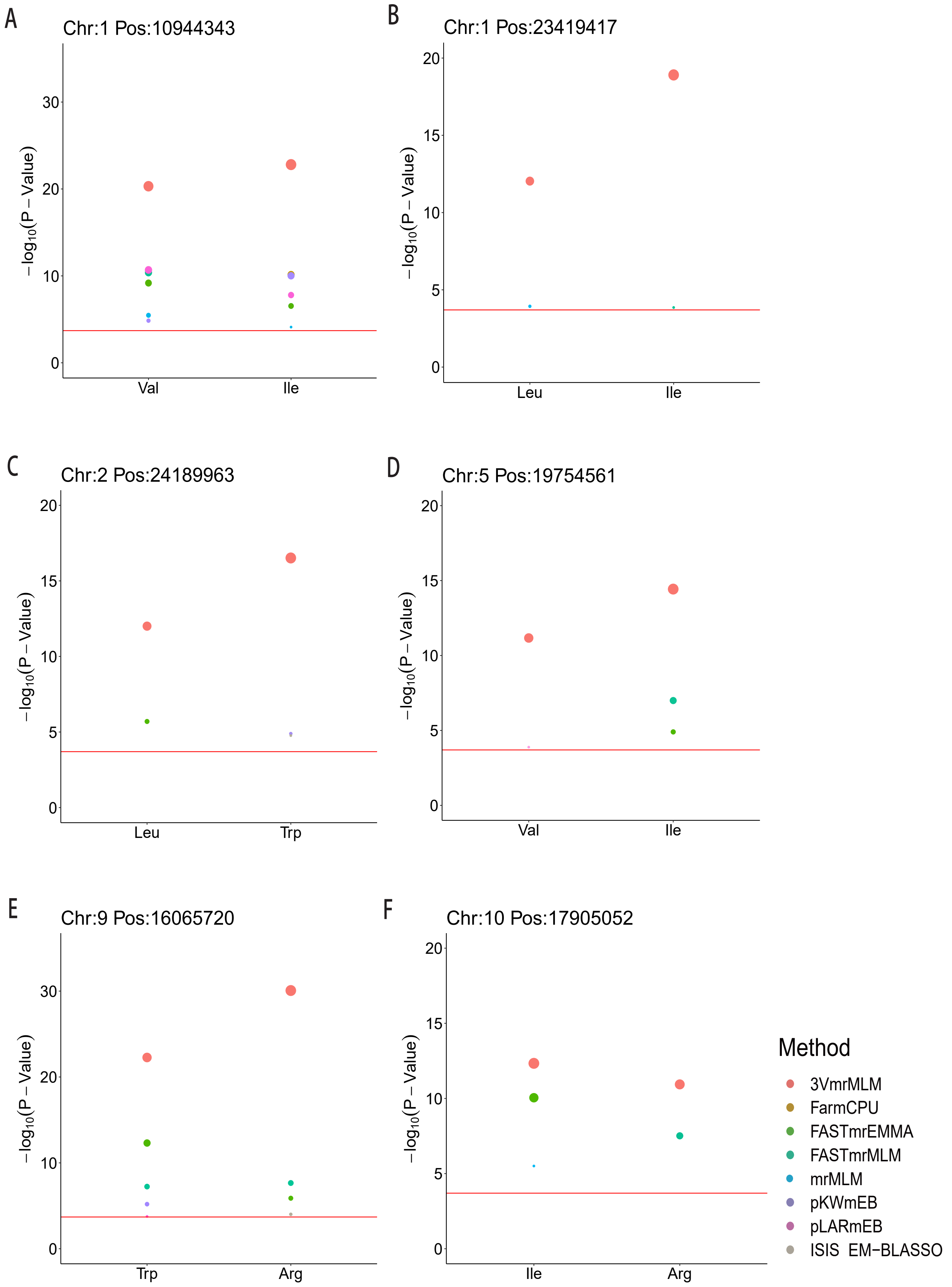

A Supplementary Figure 4

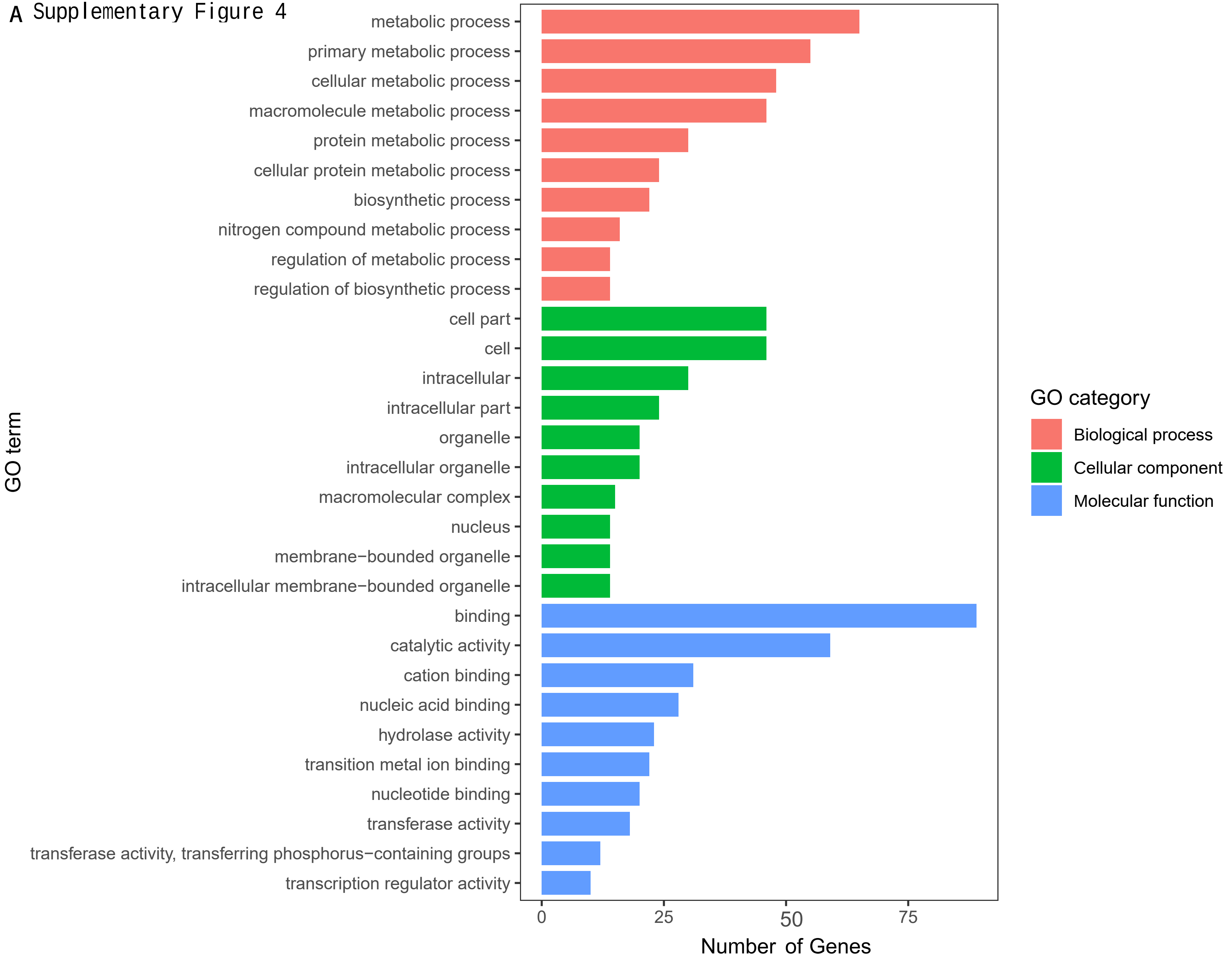

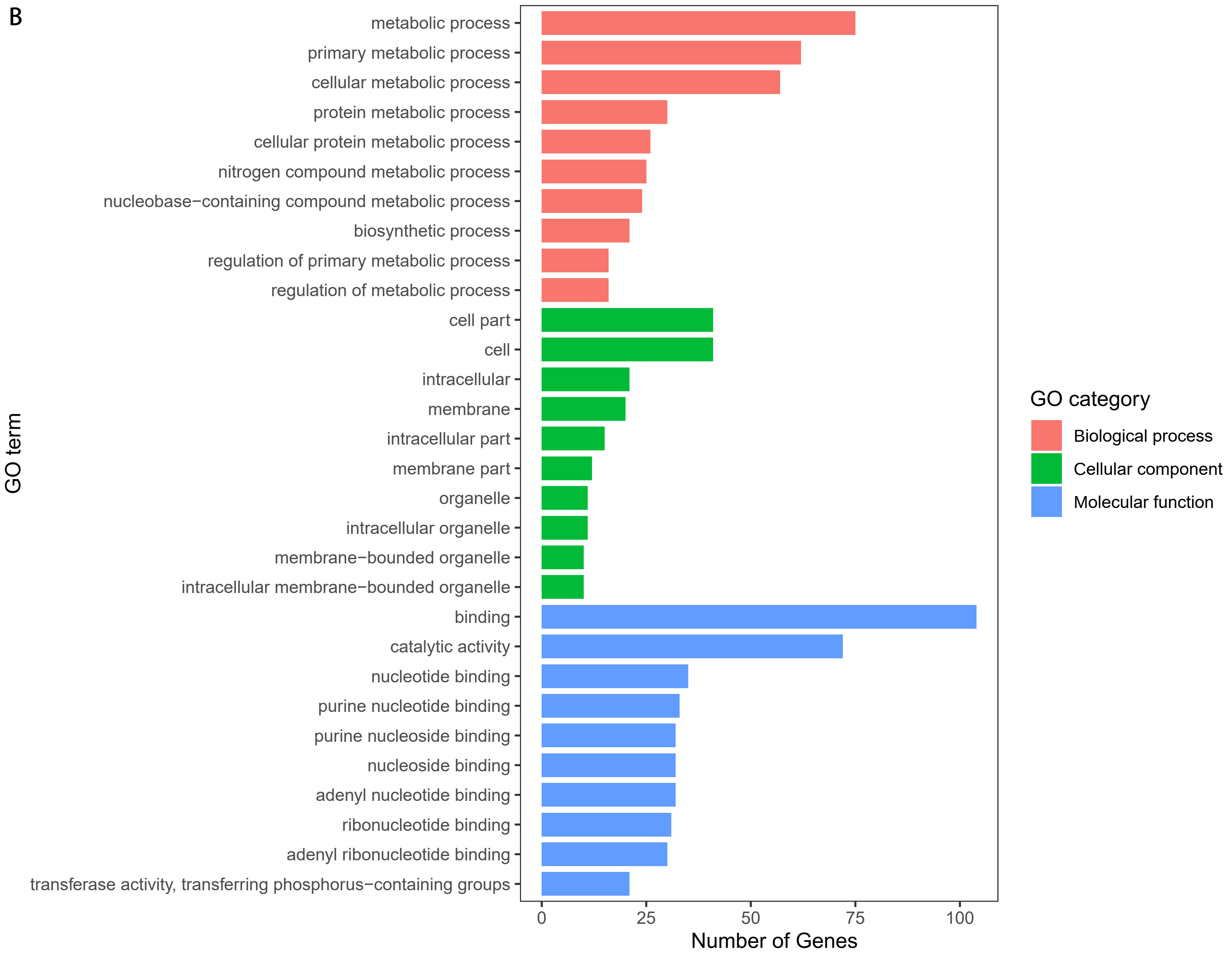
B
metabolic process
primary metabolic process
cellular metabolic process
protein metabolic process
cellular protein metabolic process
nitrogen compound metabolic process
nucleobase−containing compound metabolic process
biosynthetic process
regulation of primary metabolic process
regulation of metabolic process
cell part
cell
intracellular
membrane
intracellular part
membrane part
organelle
intracellular organelle
membrane−bounded organelle
intracellular membrane−bounded organelle
binding
catalytic activity
nucleotide binding
purine nucleotide binding
purine nucleoside binding
nucleoside binding
adenyl nucleotide binding
ribonucleotide binding
adenyl ribonucleotide binding
transferase activity, transferring phosphorus−containing groups
GO term
0
25
50
75
100
Number of Genes
GO category
Biological process
Cellular component
Molecular function

C

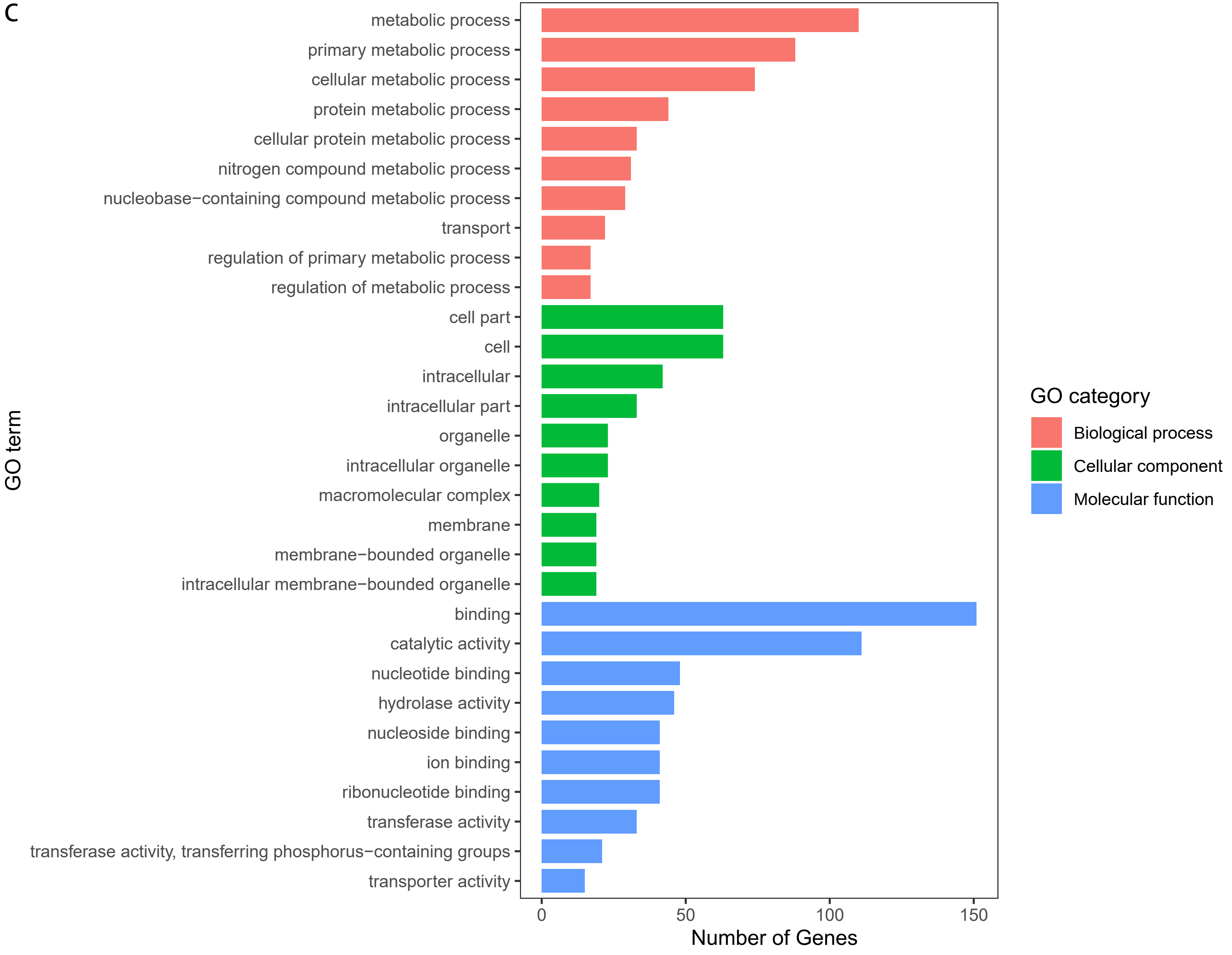
metabolic process
primary metabolic process
cellular metabolic process
protein metabolic process
cellular protein metabolic process
nitrogen compound metabolic process
nucleobase−containing compound metabolic process
transport
regulation of primary metabolic process
regulation of metabolic process
cell part
cell
intracellular
intracellular part
organelle
intracellular organelle
macromolecular complex
membrane
membrane−bounded organelle
intracellular membrane−bounded organelle
binding
catalytic activity
nucleotide binding
hydrolase activity
nucleoside binding
ion binding
ribonucleotide binding
transferase activity
transferase activity, transferring phosphorus−containing groups
transporter activity
GO term
0
50
100
150
Number of Genes
GO category
Biological process
Cellular component
Molecular function

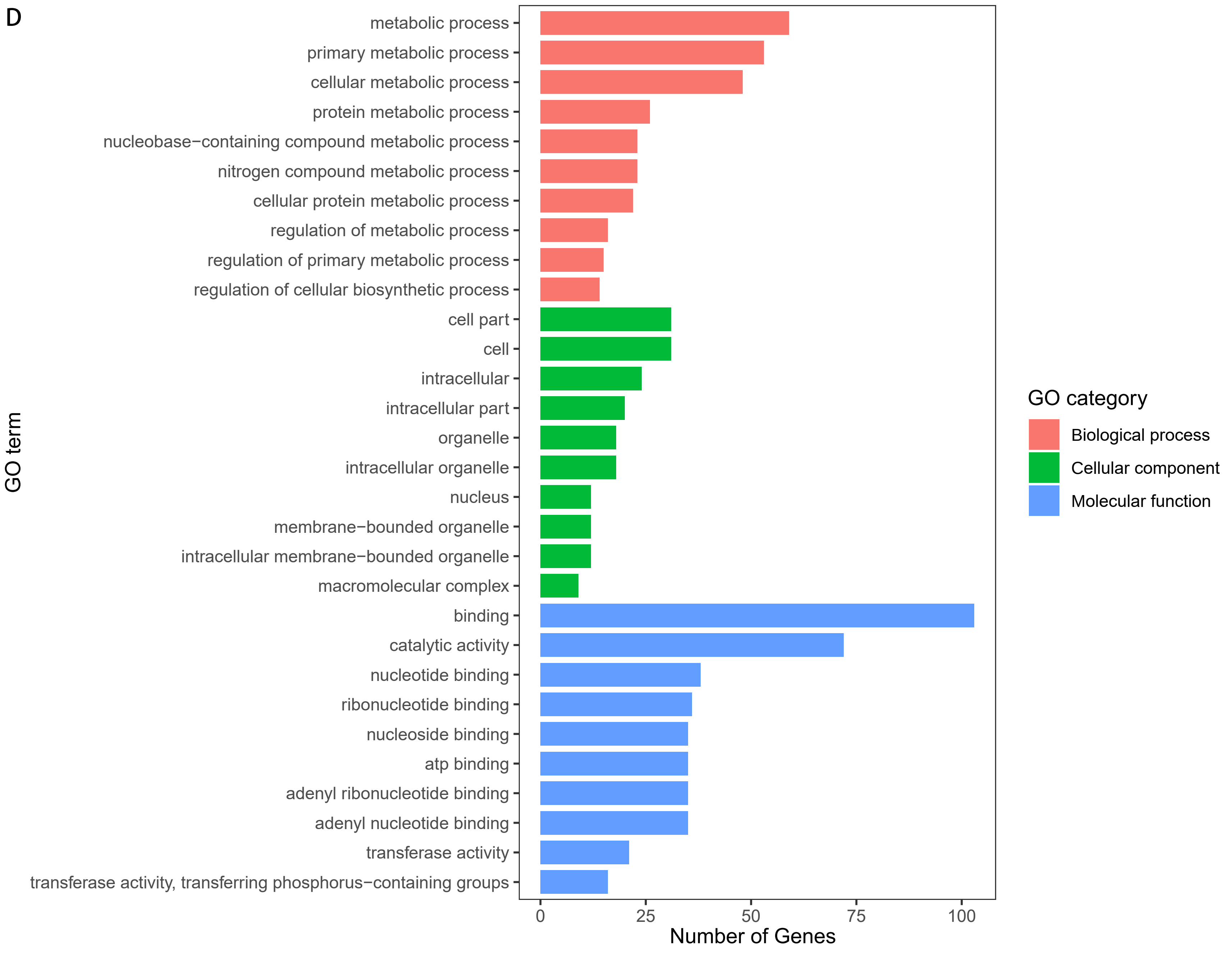
D
metabolic process
primary metabolic process
cellular metabolic process
protein metabolic process
nucleobase−containing compound metabolic process
nitrogen compound metabolic process
cellular protein metabolic process
regulation of metabolic process
regulation of primary metabolic process
regulation of cellular biosynthetic process
cell part
cell
intracellular
intracellular part
organelle
intracellular organelle
nucleus
membrane−bounded organelle
intracellular membrane−bounded organelle
macromolecular complex
binding
catalytic activity
nucleotide binding
ribonucleotide binding
nucleoside binding
atp binding
adenyl ribonucleotide binding
adenyl nucleotide binding
transferase activity
transferase activity, transferring phosphorus−containing groups
GO term
0
25
50
75
100
Number of Genes
GO category
Biological process
Cellular component
Molecular function

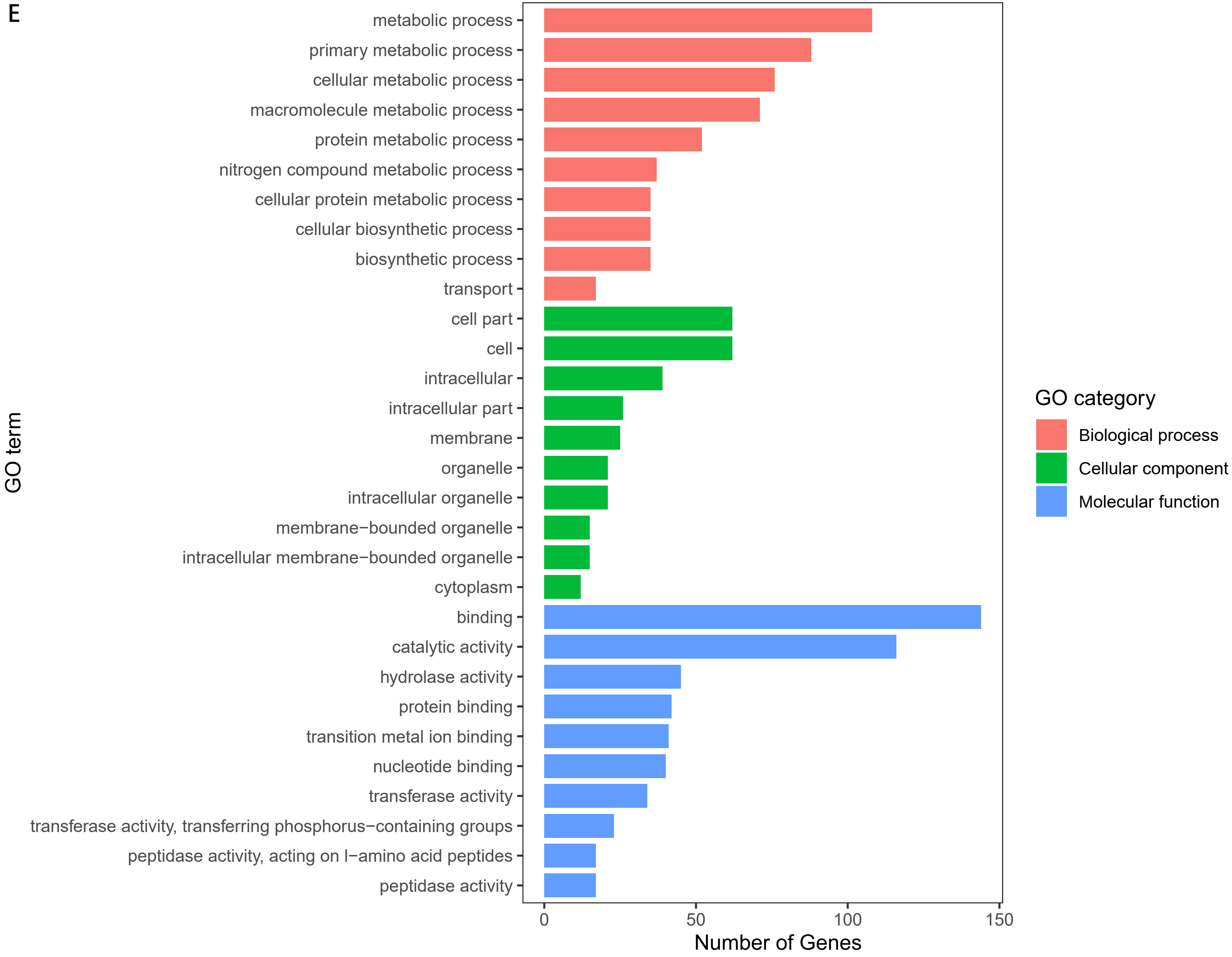

E
metabolic process
primary metabolic process
cellular metabolic process
macromolecule metabolic process
protein metabolic process
nitrogen compound metabolic process
cellular protein metabolic process
cellular biosynthetic process
biosynthetic process
transport
cell part
cell
intracellular
intracellular part
membrane
organelle
intracellular organelle
membrane−bounded organelle
intracellular membrane−bounded organelle
cytoplasm
binding
catalytic activity
hydrolase activity
protein binding
transition metal ion binding
nucleotide binding
transferase activity
transferase activity, transferring phosphorus−containing groups
peptidase activity, acting on l−amino acid peptides
peptidase activity
GO term
0
50
100
150
Number of Genes
GO category
Biological process
Cellular component
Molecular function

Supplementary Figure 5

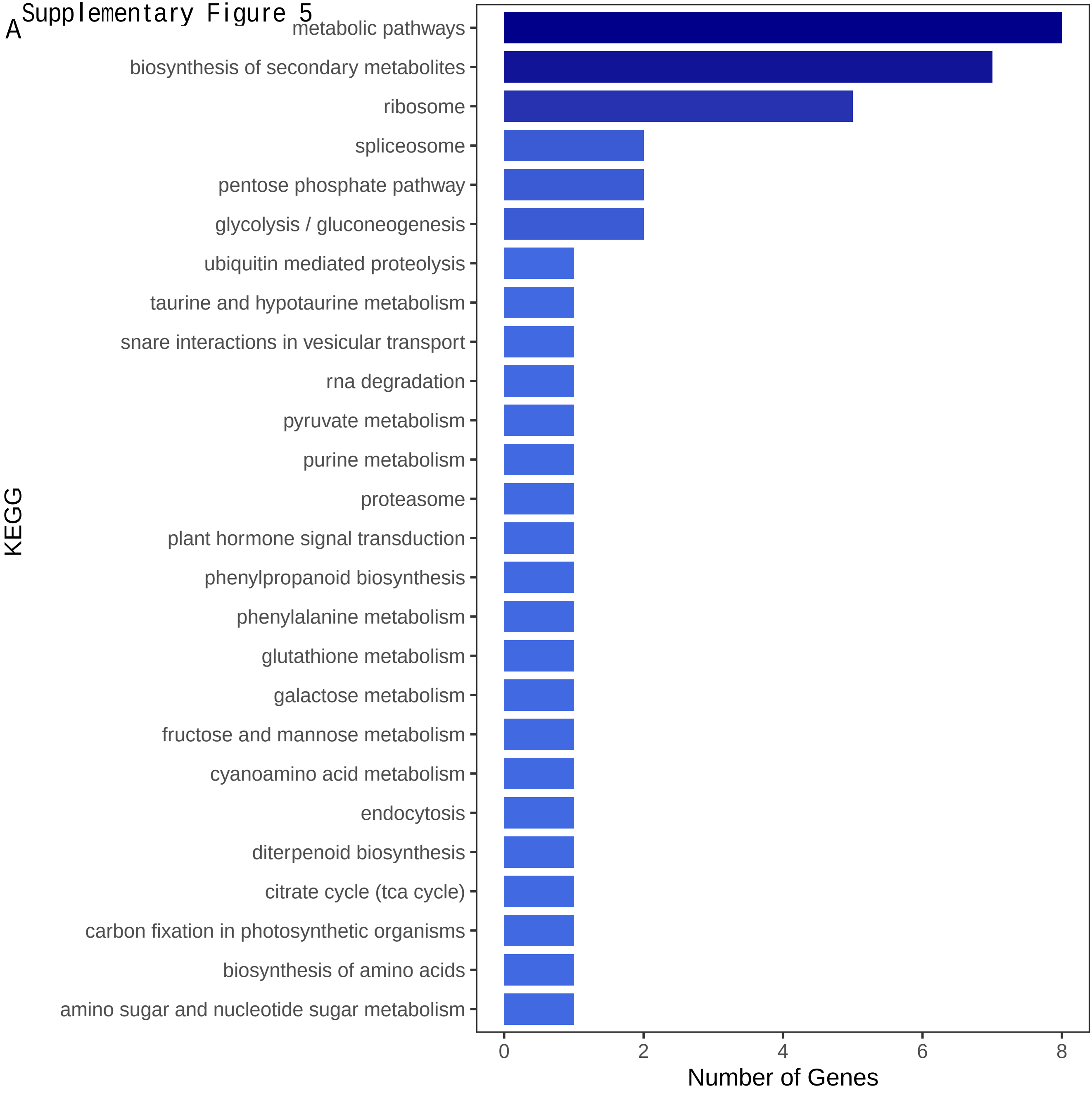

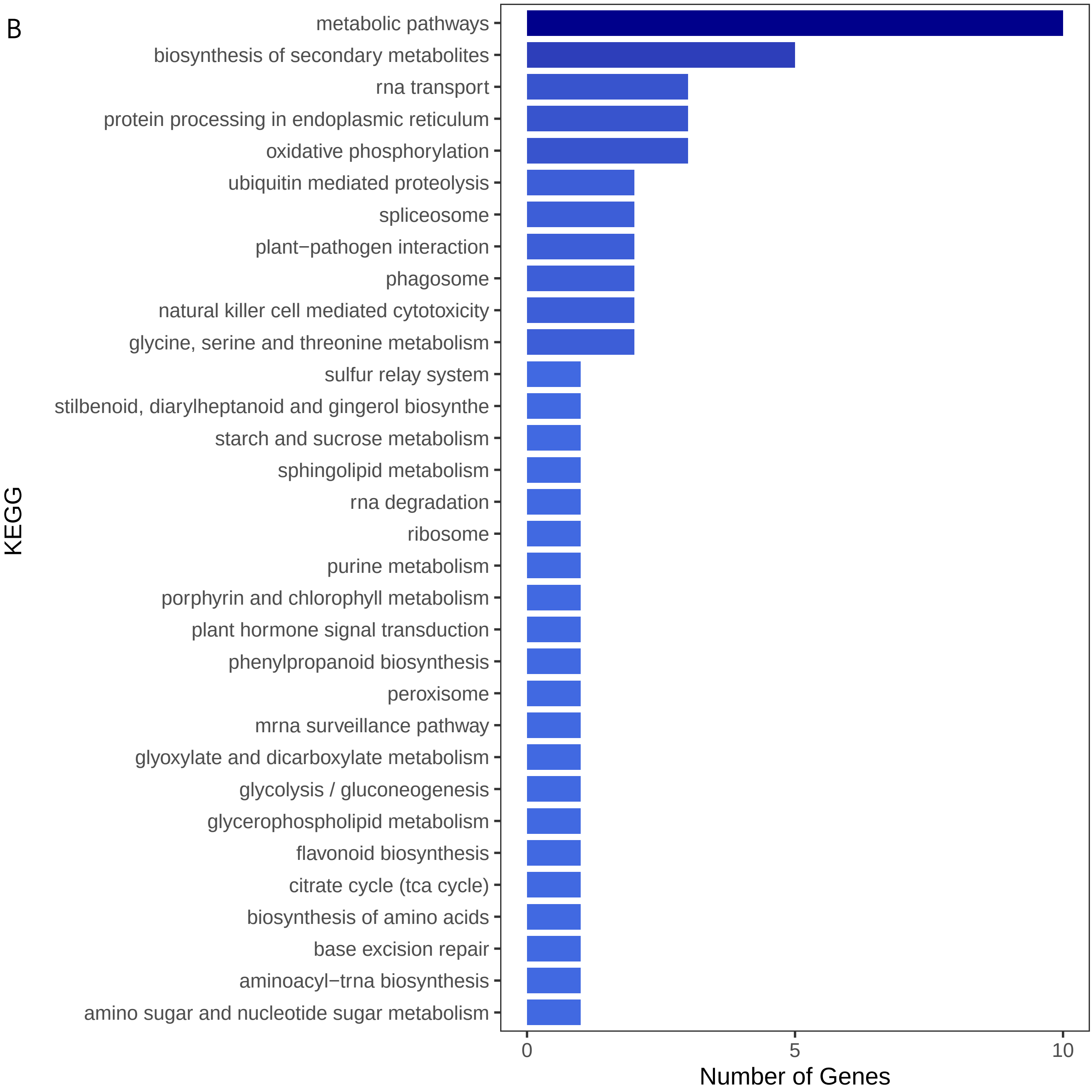
B
metabolic pathways
biosynthesis of secondary metabolites
rna transport
protein processing in endoplasmic reticulum
oxidative phosphorylation
ubiquitin mediated proteolysis
spliceosome
plant−pathogen interaction
phagosome
natural killer cell mediated cytotoxicity
glycine, serine and threonine metabolism
sulfur relay system
stilbenoid, diarylheptanoid and gingerol biosynthe
starch and sucrose metabolism
sphingolipid metabolism
rna degradation
ribosome
purine metabolism
porphyrin and chlorophyll metabolism
plant hormone signal transduction
phenylpropanoid biosynthesis
peroxisome
mrna surveillance pathway
glyoxylate and dicarboxylate metabolism
glycolysis / gluconeogenesis
glycerophospholipid metabolism
flavonoid biosynthesis
citrate cycle (tca cycle)
biosynthesis of amino acids
base excision repair
aminoacyl−trna biosynthesis
amino sugar and nucleotide sugar metabolism
KEGG
0
5
10
Number of Genes

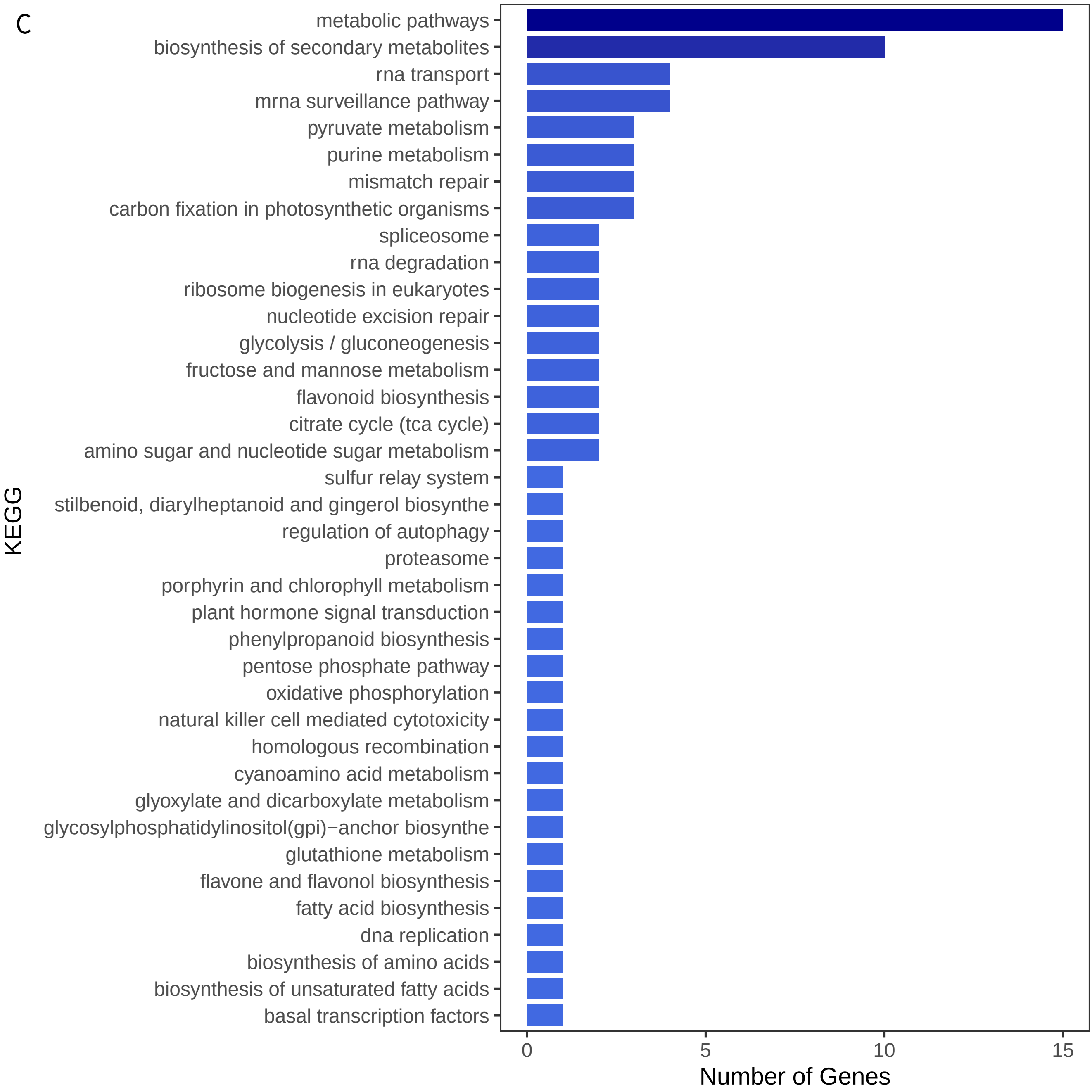
C
metabolic pathways
biosynthesis of secondary metabolites
rna transport
mrna surveillance pathway
pyruvate metabolism
purine metabolism
mismatch repair
carbon fixation in photosynthetic organisms
spliceosome
rna degradation
ribosome biogenesis in eukaryotes
nucleotide excision repair
glycolysis / gluconeogenesis
fructose and mannose metabolism
flavonoid biosynthesis
citrate cycle (tca cycle)
amino sugar and nucleotide sugar metabolism
sulfur relay system
stilbenoid, diarylheptanoid and gingerol biosynthe
regulation of autophagy
proteasome
porphyrin and chlorophyll metabolism
plant hormone signal transduction
phenylpropanoid biosynthesis
pentose phosphate pathway
oxidative phosphorylation
natural killer cell mediated cytotoxicity
homologous recombination
cyanoamino acid metabolism
glyoxylate and dicarboxylate metabolism
glycosylphosphatidylinositol(gpi)−anchor biosynthe
glutathione metabolism
flavone and flavonol biosynthesis
fatty acid biosynthesis
dna replication
biosynthesis of amino acids
biosynthesis of unsaturated fatty acids
basal transcription factors
KEGG
0
5
10
15
Number of Genes

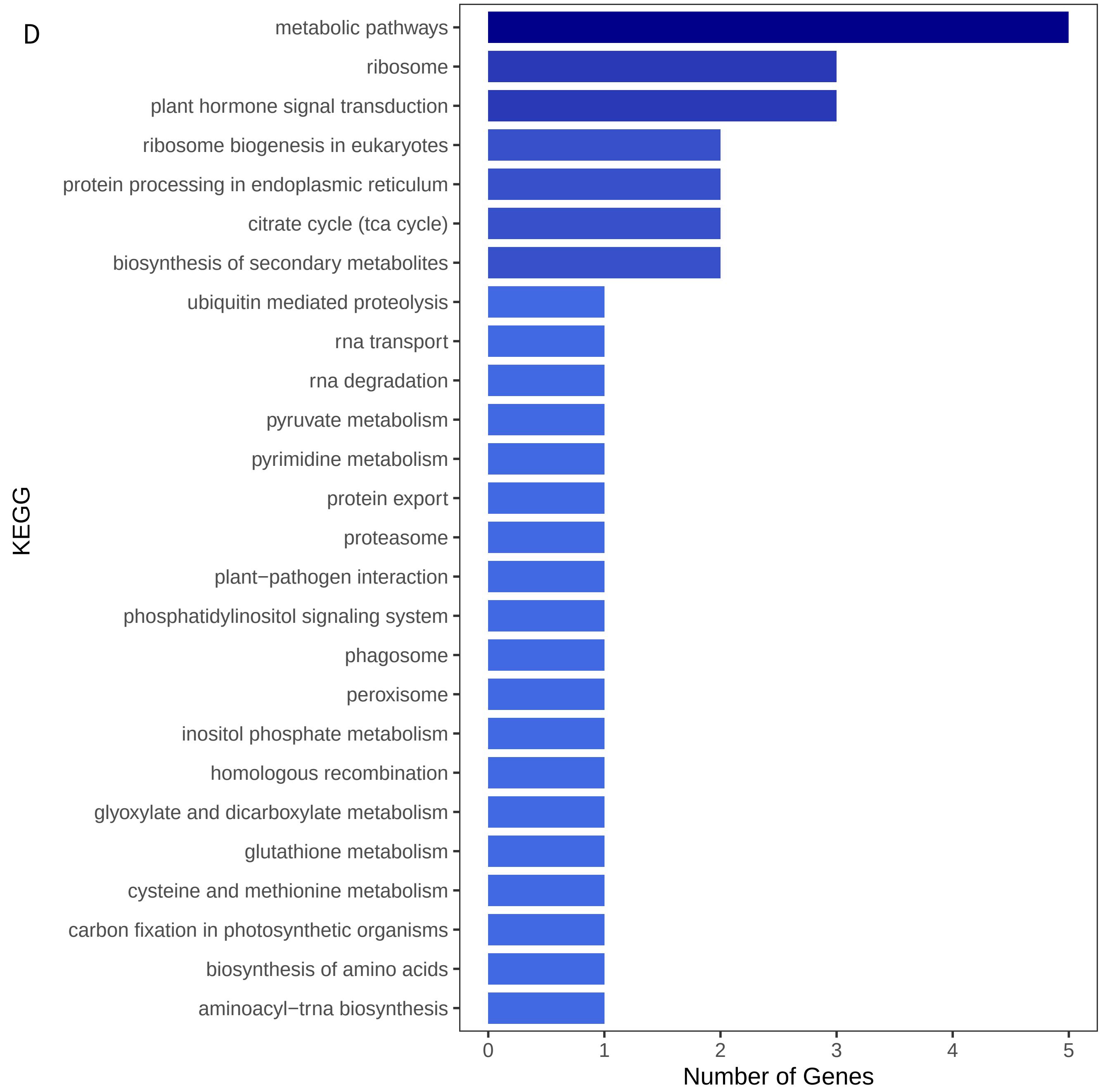

D
metabolic pathways
ribosome
plant hormone signal transduction
ribosome biogenesis in eukaryotes
protein processing in endoplasmic reticulum
citrate cycle (tca cycle)
biosynthesis of secondary metabolites
ubiquitin mediated proteolysis
rna transport
rna degradation
pyruvate metabolism
pyrimidine metabolism
protein export
proteasome
plant−pathogen interaction
phosphatidylinositol signaling system
phagosome
peroxisome
inositol phosphate metabolism
homologous recombination
glyoxylate and dicarboxylate metabolism
glutathione metabolism
cysteine and methionine metabolism
carbon fixation in photosynthetic organisms
biosynthesis of amino acids
aminoacyl−trna biosynthesis
KEGG
0
1
2
3
4
5
Number of Genes

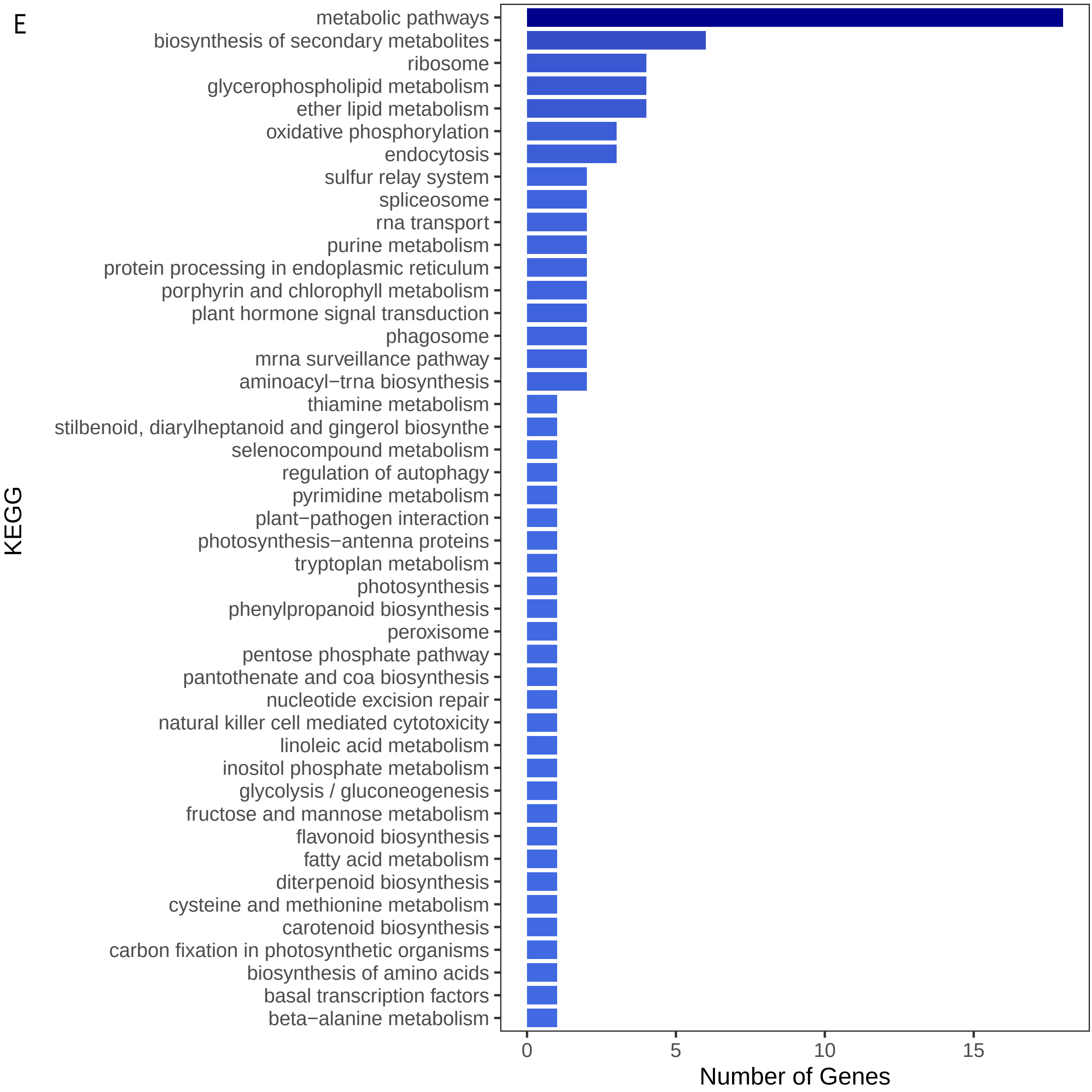
E
KEGG
metabolic pathways
biosynthesis of secondary metabolites
ribosome
glycerophospholipid metabolism
ether lipid metabolism
oxidative phosphorylation
endocytosis
sulfur relay system
spliceosome
rna transport
purine metabolism
protein processing in endoplasmic reticulum
porphyrin and chlorophyll metabolism
plant hormone signal transduction
phagosome
mrna surveillance pathway
aminoacyl−trna biosynthesis
thiamine metabolism
stilbenoid, diarylheptanoid and gingerol biosynthe
selenocompound metabolism
regulation of autophagy
pyrimidine metabolism
plant−pathogen interaction
photosynthesis−antenna proteins
tryptoplan metabolism
photosynthesis
phenylpropanoid biosynthesis
peroxisome
pentose phosphate pathway
pantothenate and coa biosynthesis
nucleotide excision repair
natural killer cell mediated cytotoxicity
linoleic acid metabolism
inositol phosphate metabolism
glycolysis / gluconeogenesis
fructose and mannose metabolism
flavonoid biosynthesis
fatty acid metabolism
diterpenoid biosynthesis
cysteine and methionine metabolism
carotenoid biosynthesis
carbon fixation in photosynthetic organisms
biosynthesis of amino acids
basal transcription factors
beta−alanine metabolism
0
5
10
15
Number of Genes

Supplementary Figure 6

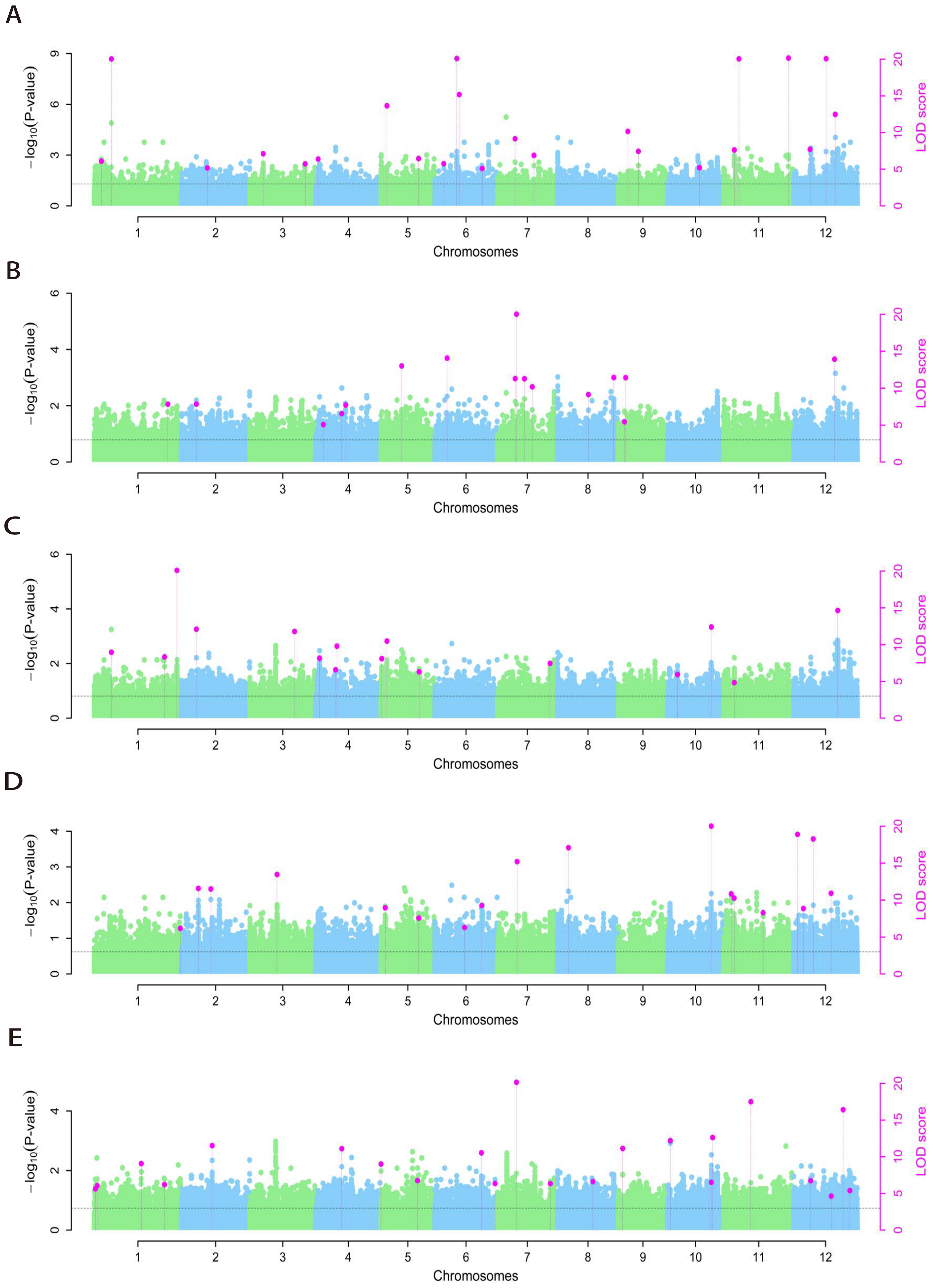

A Supplementary Figure 7

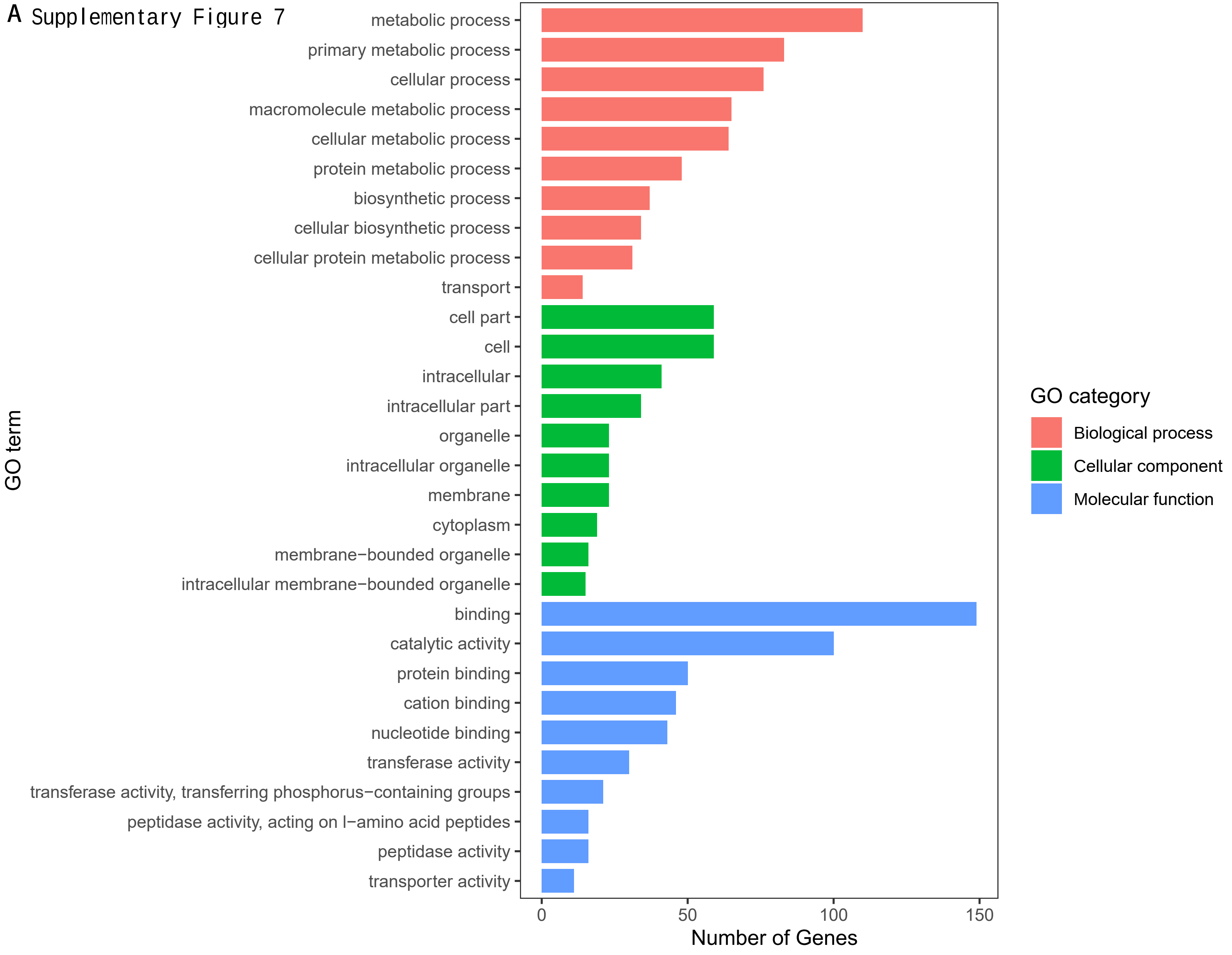

B

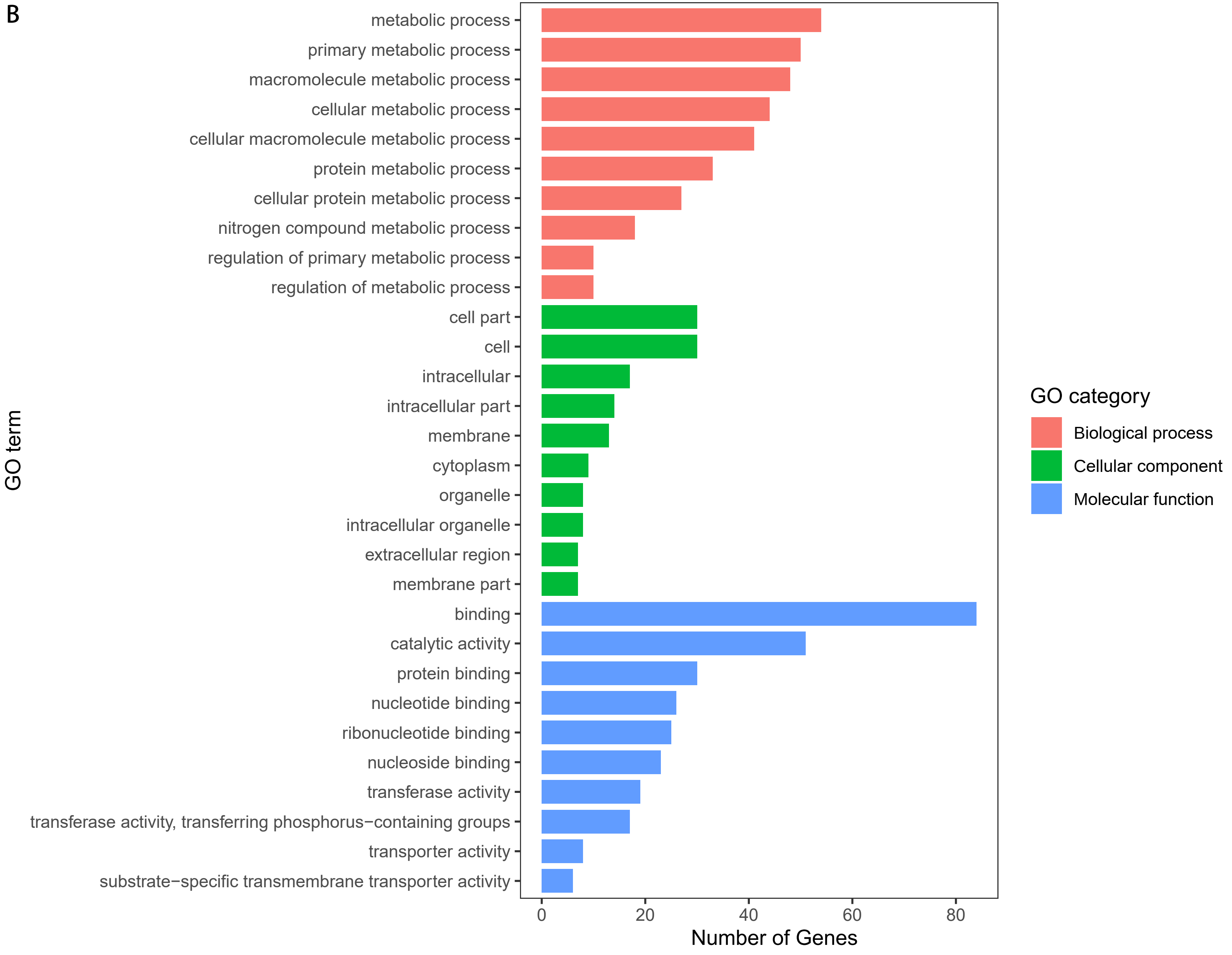

metabolic process
primary metabolic process
macromolecule metabolic process
cellular metabolic process
cellular macromolecule metabolic process
protein metabolic process
cellular protein metabolic process
nitrogen compound metabolic process
regulation of primary metabolic process
regulation of metabolic process
cell part
cell
intracellular
intracellular part
membrane
cytoplasm
organelle
intracellular organelle
extracellular region
membrane part
binding
catalytic activity
protein binding
nucleotide binding
ribonucleotide binding
nucleoside binding
transferase activity
transferase activity, transferring phosphorus−containing groups
transporter activity
substrate−specific transmembrane transporter activity
GO term
0
20
40
60
80
Number of Genes
GO category
Biological process
Cellular component
Molecular function

C

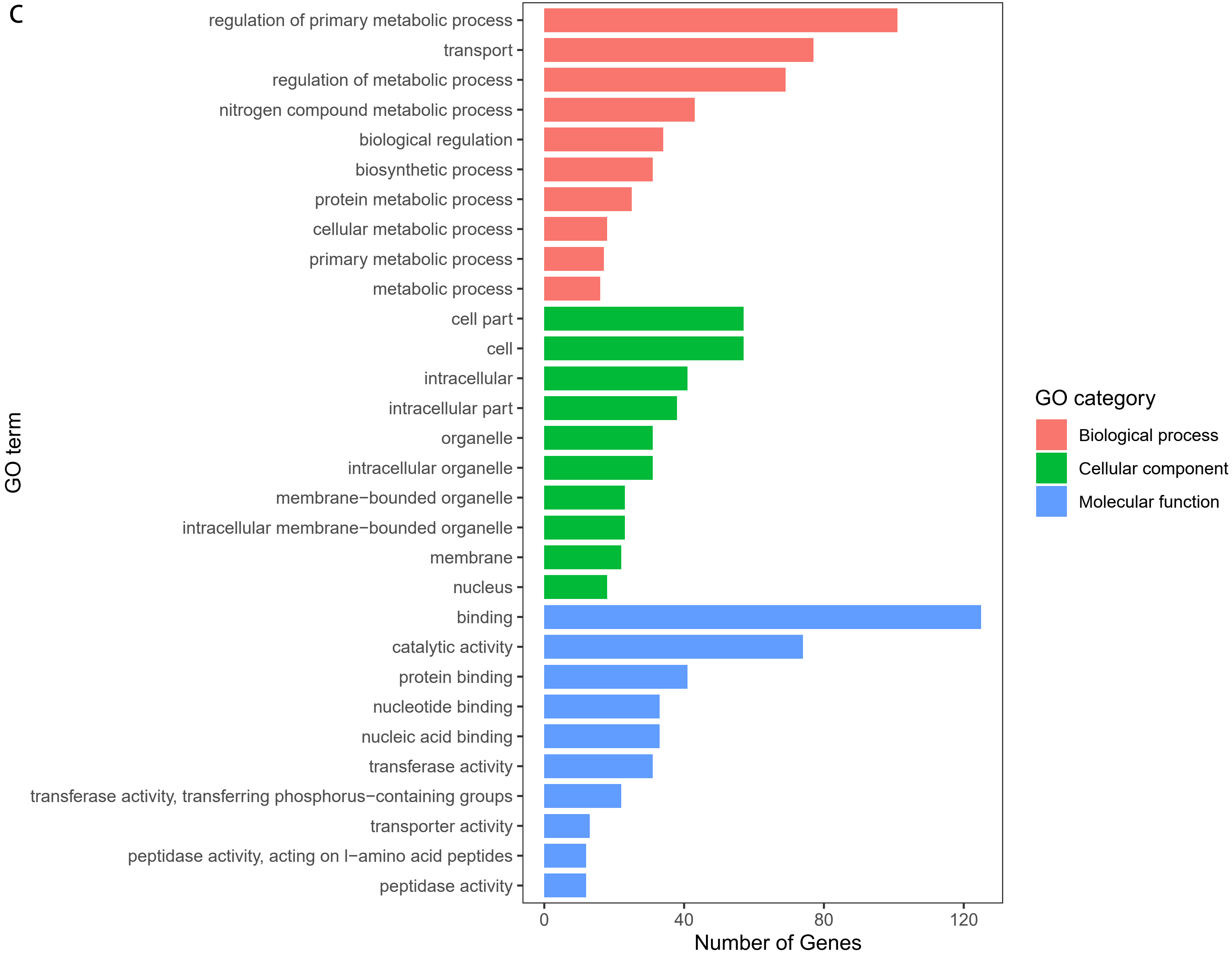

regulation of primary metabolic process
transport
regulation of metabolic process
nitrogen compound metabolic process
biological regulation
biosynthetic process
protein metabolic process
cellular metabolic process
primary metabolic process
metabolic process
cell part
cell
intracellular
intracellular part
organelle
intracellular organelle
membrane−bounded organelle
intracellular membrane−bounded organelle
membrane
nucleus
binding
catalytic activity
protein binding
nucleotide binding
nucleic acid binding
transferase activity
transferase activity, transferring phosphorus−containing groups
transporter activity
peptidase activity, acting on l−amino acid peptides
peptidase activity
GO term
0
40
80
120
Number of Genes
GO category
Biological process
Cellular component
Molecular function

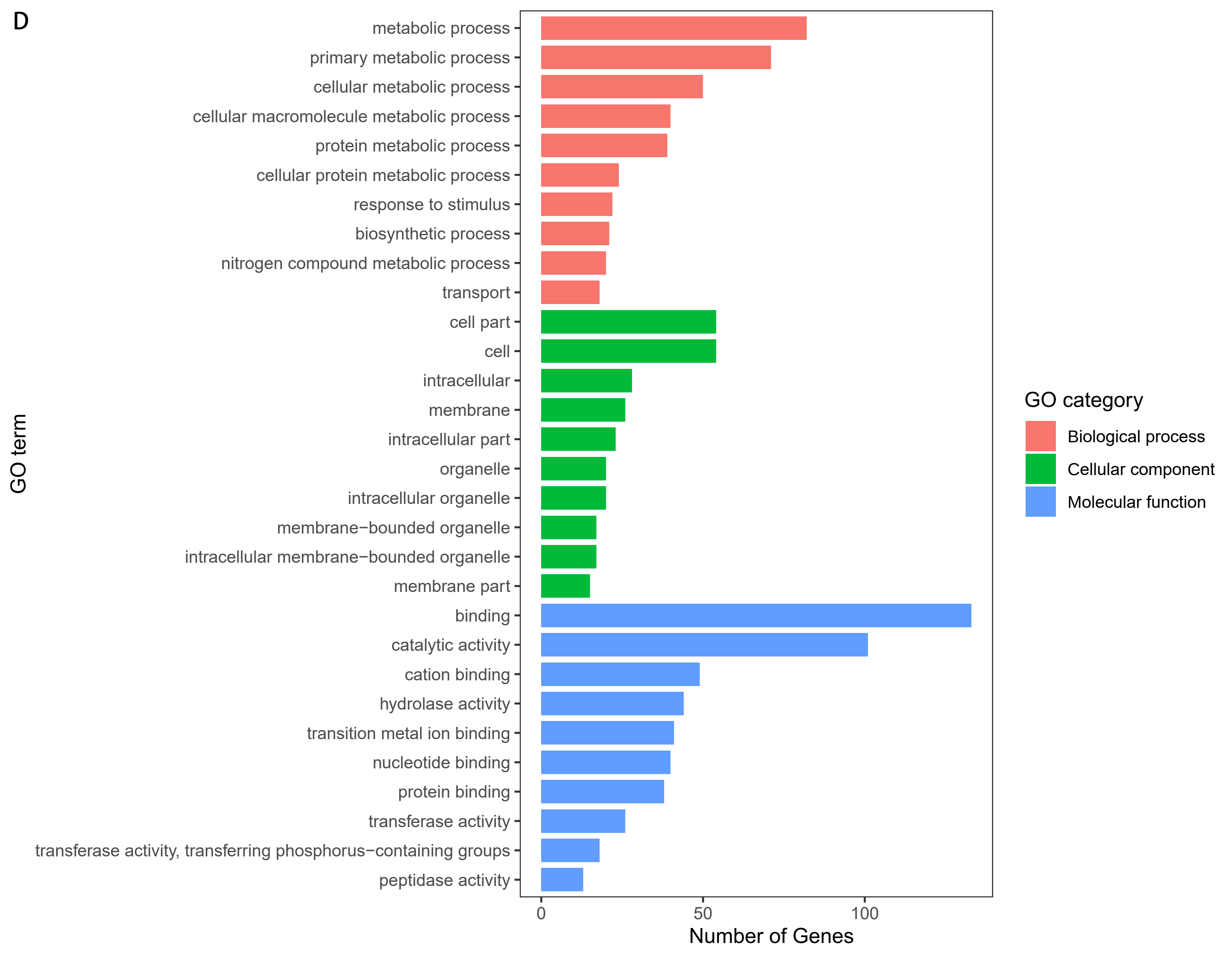
D
GO term
metabolic process
primary metabolic process
cellular metabolic process
cellular macromolecule metabolic process
protein metabolic process
cellular protein metabolic process
response to stimulus
biosynthetic process
nitrogen compound metabolic process
transport
cell part
cell
intracellular
membrane
intracellular part
organelle
intracellular organelle
membrane−bounded organelle
intracellular membrane−bounded organelle
membrane part
binding
catalytic activity
cation binding
hydrolase activity
transition metal ion binding
nucleotide binding
protein binding
transferase activity
transferase activity, transferring phosphorus−containing groups
peptidase activity
0
50
100
Number of Genes
GO category
Biological process
Cellular component
Molecular function

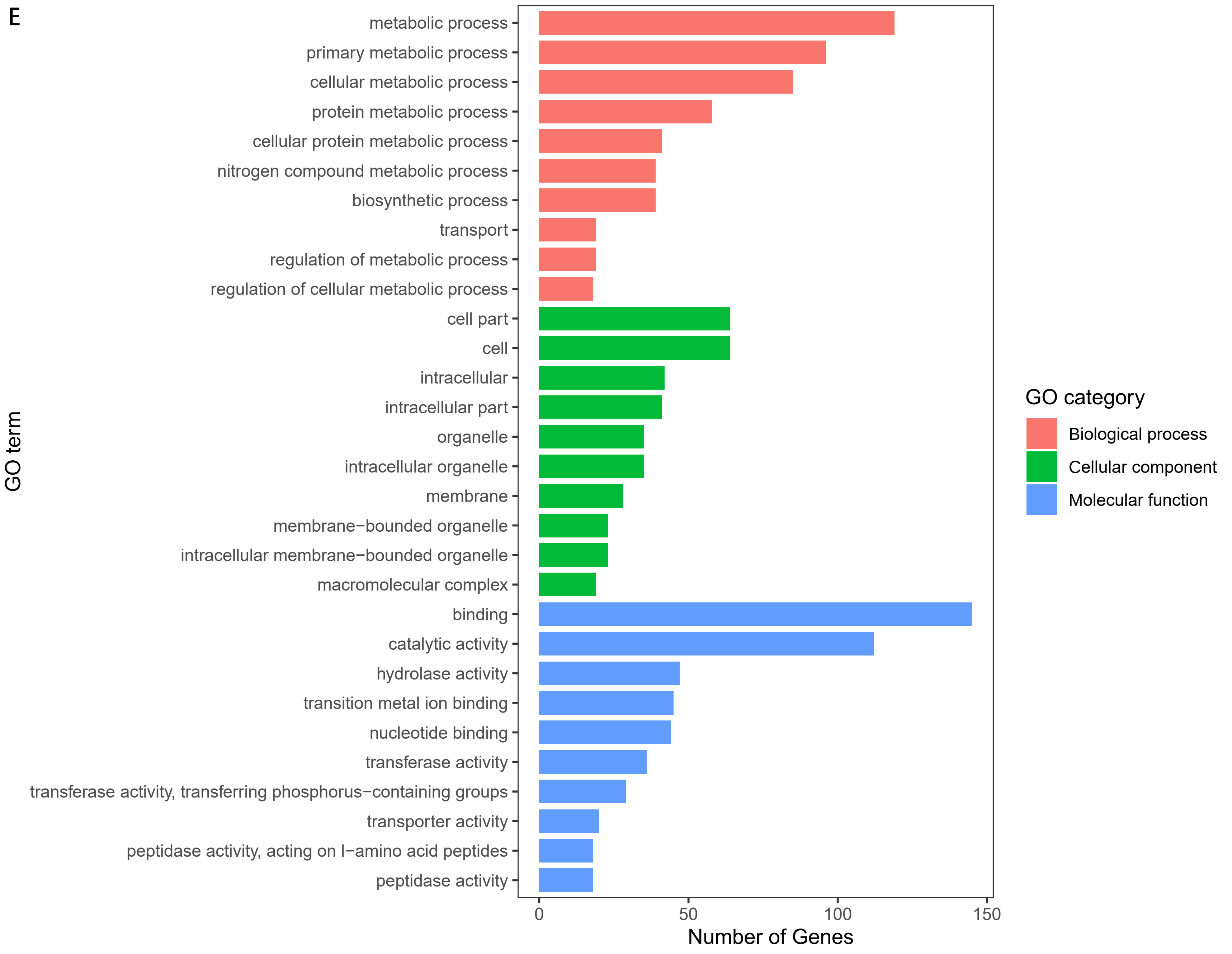

E
metabolic process
primary metabolic process
cellular metabolic process
protein metabolic process
cellular protein metabolic process
nitrogen compound metabolic process
biosynthetic process
transport
regulation of metabolic process
regulation of cellular metabolic process
cell part
cell
intracellular
intracellular part
organelle
intracellular organelle
membrane
membrane−bounded organelle
intracellular membrane−bounded organelle
macromolecular complex
binding
catalytic activity
hydrolase activity
transition metal ion binding
nucleotide binding
transferase activity
transferase activity, transferring phosphorus−containing groups
transporter activity
peptidase activity, acting on l−amino acid peptides
peptidase activity
GO term
0
50
100
150
Number of Genes
GO category
Biological process
Cellular component
Molecular function

A Supplementary Figure 8

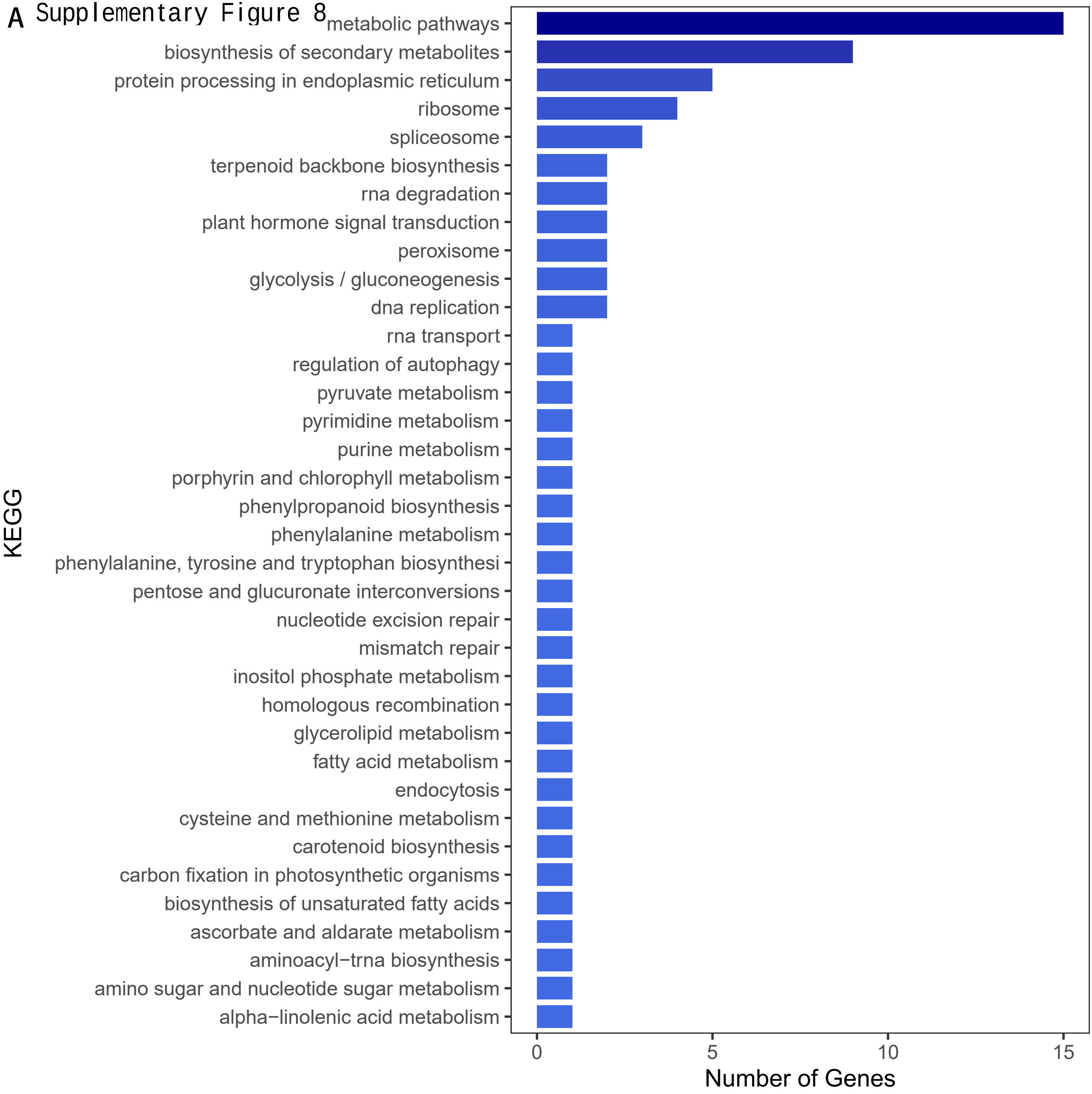

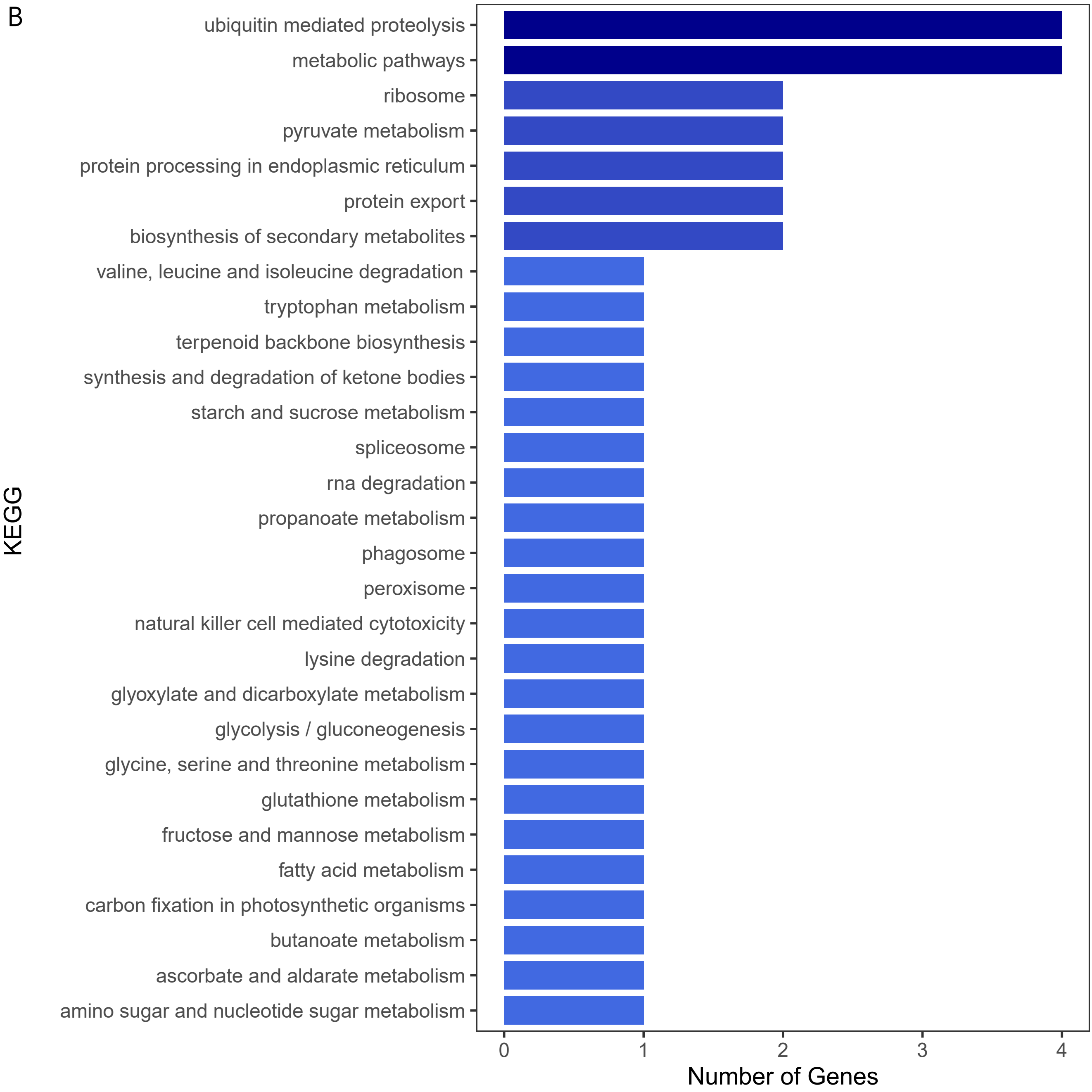
B
ubiquitin mediated proteolysis
metabolic pathways
ribosome
pyruvate metabolism
protein processing in endoplasmic reticulum
protein export
biosynthesis of secondary metabolites
valine, leucine and isoleucine degradation
tryptophan metabolism
terpenoid backbone biosynthesis
synthesis and degradation of ketone bodies
starch and sucrose metabolism
spliceosome
rna degradation
propanoate metabolism
phagosome
peroxisome
natural killer cell mediated cytotoxicity
lysine degradation
glyoxylate and dicarboxylate metabolism
glycolysis / gluconeogenesis
glycine, serine and threonine metabolism
glutathione metabolism
fructose and mannose metabolism
fatty acid metabolism
carbon fixation in photosynthetic organisms
butanoate metabolism
ascorbate and aldarate metabolism
amino sugar and nucleotide sugar metabolism
KEGG
0
1
2
3
4
Number of Genes

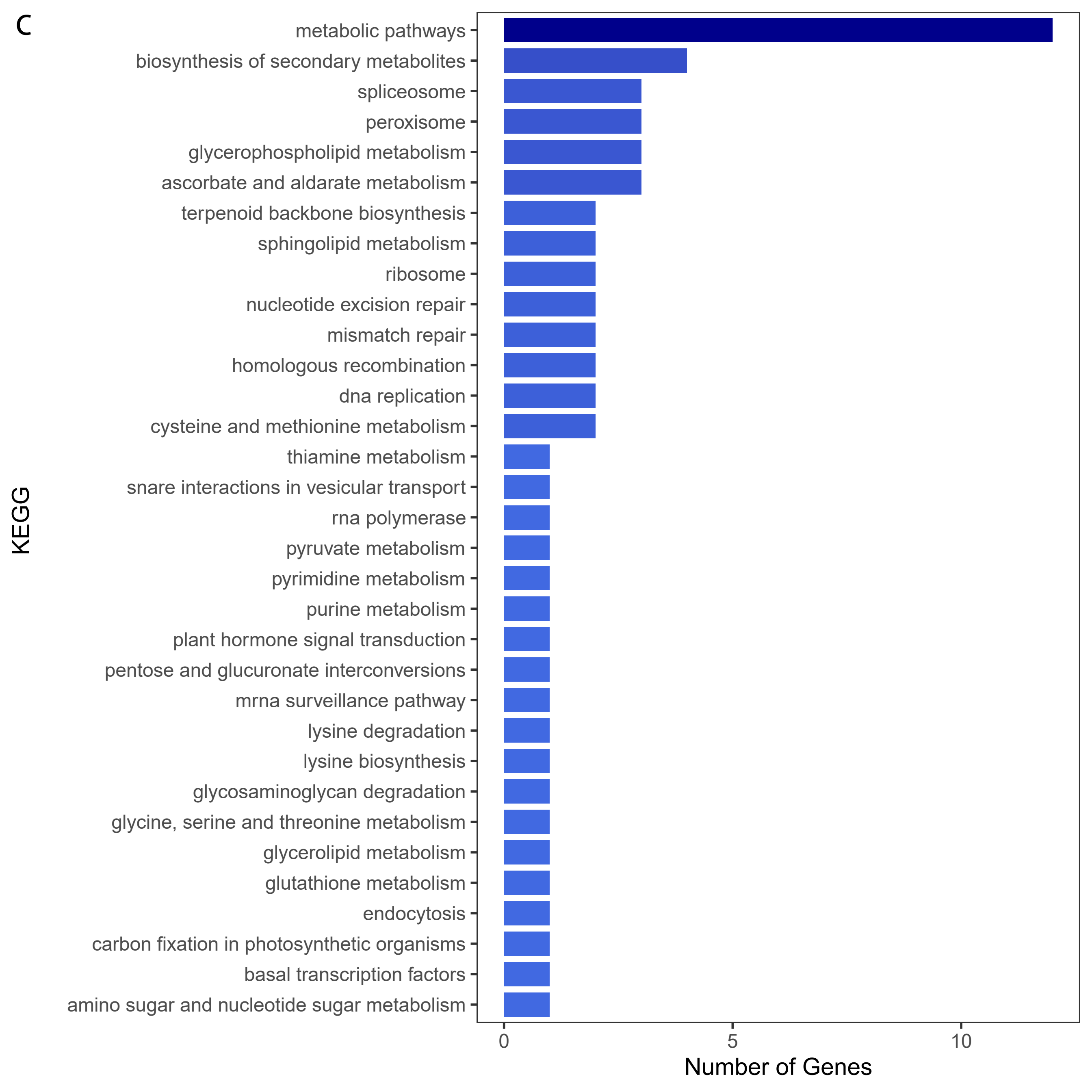
C
metabolic pathways
biosynthesis of secondary metabolites
spliceosome
peroxisome
glycerophospholipid metabolism
ascorbate and aldarate metabolism
terpenoid backbone biosynthesis
sphingolipid metabolism
ribosome
nucleotide excision repair
mismatch repair
homologous recombination
dna replication
cysteine and methionine metabolism
thiamine metabolism
snare interactions in vesicular transport
rna polymerase
pyruvate metabolism
pyrimidine metabolism
purine metabolism
plant hormone signal transduction
pentose and glucuronate interconversions
mrna surveillance pathway
lysine degradation
lysine biosynthesis
glycosaminoglycan degradation
glycine, serine and threonine metabolism
glycerolipid metabolism
glutathione metabolism
endocytosis
carbon fixation in photosynthetic organisms
basal transcription factors
amino sugar and nucleotide sugar metabolism
KEGG
0
5
10
Number of Genes

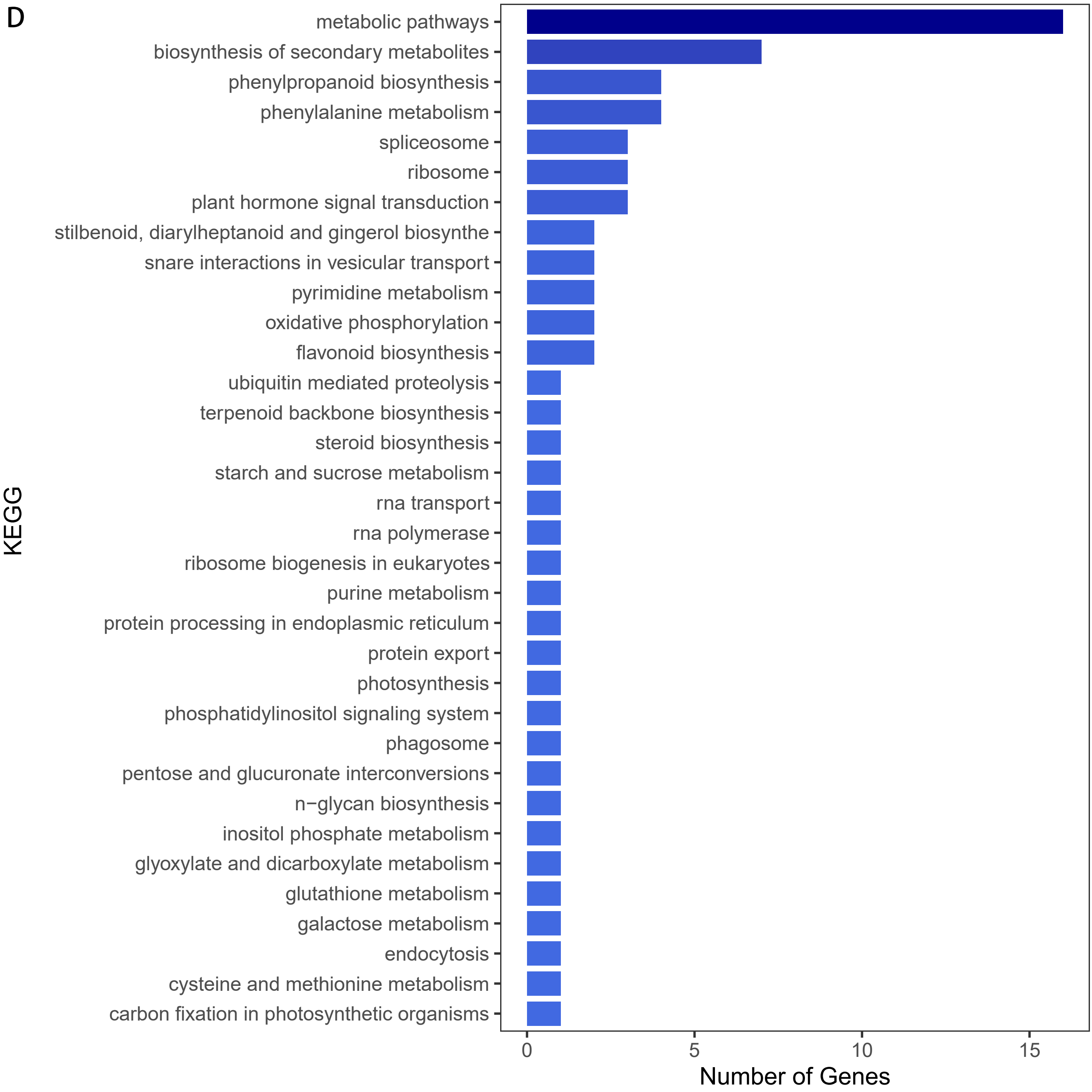
D
metabolic pathways
biosynthesis of secondary metabolites
phenylpropanoid biosynthesis
phenylalanine metabolism
spliceosome
ribosome
plant hormone signal transduction
stilbenoid, diarylheptanoid and gingerol biosynthe
snare interactions in vesicular transport
pyrimidine metabolism
oxidative phosphorylation
flavonoid biosynthesis
ubiquitin mediated proteolysis
terpenoid backbone biosynthesis
steroid biosynthesis
starch and sucrose metabolism
rna transport
rna polymerase
ribosome biogenesis in eukaryotes
purine metabolism
protein processing in endoplasmic reticulum
protein export
photosynthesis
phosphatidylinositol signaling system
phagosome
pentose and glucuronate interconversions
n−glycan biosynthesis
inositol phosphate metabolism
glyoxylate and dicarboxylate metabolism
glutathione metabolism
galactose metabolism
endocytosis
cysteine and methionine metabolism
carbon fixation in photosynthetic organisms
KEGG
0
5
10
15
Number of Genes

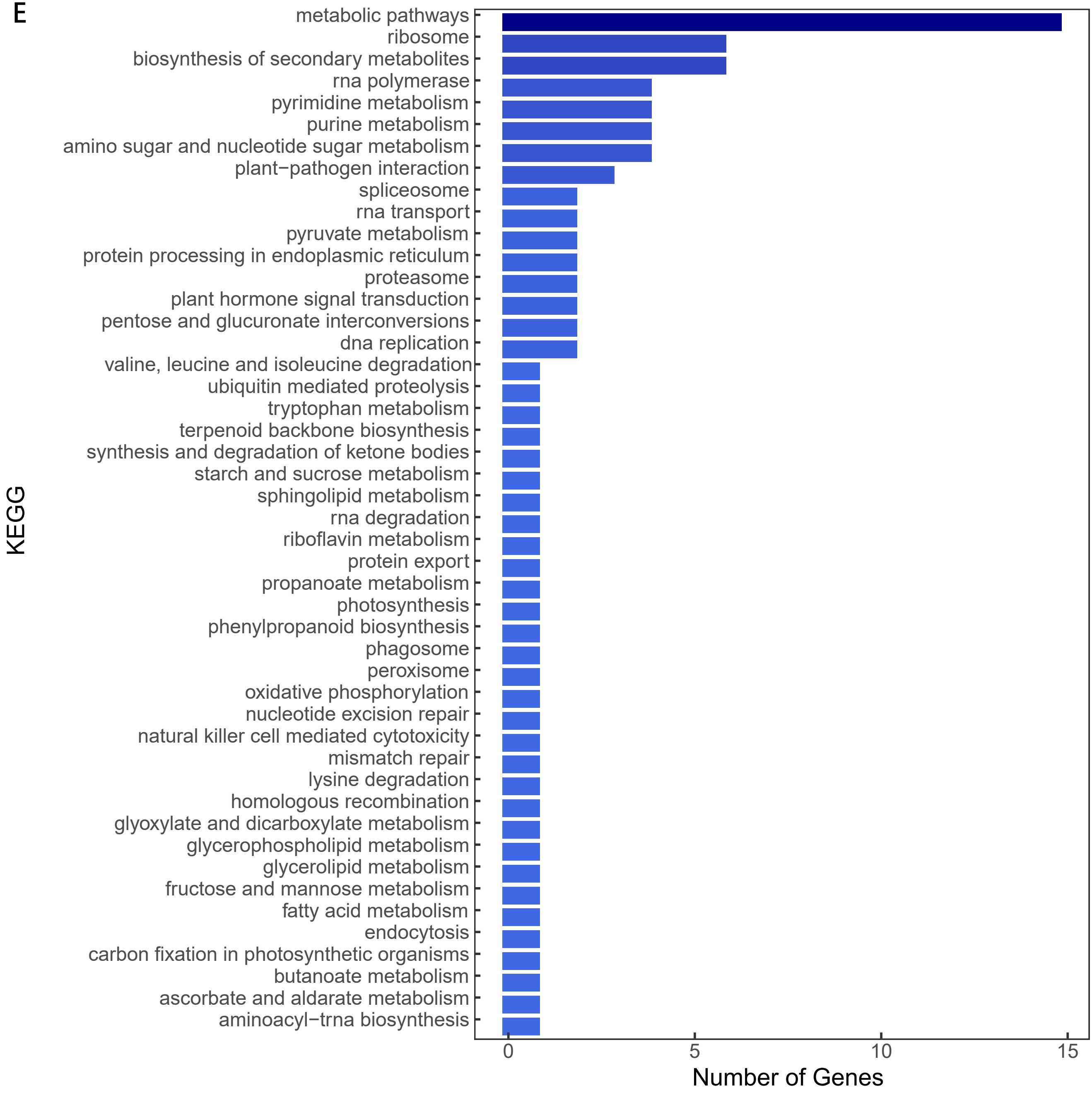
E
metabolic pathways
ribosome
biosynthesis of secondary metabolites
rna polymerase
pyrimidine metabolism
purine metabolism
amino sugar and nucleotide sugar metabolism
plant−pathogen interaction
spliceosome
rna transport
pyruvate metabolism
protein processing in endoplasmic reticulum
proteasome
plant hormone signal transduction
pentose and glucuronate interconversions
dna replication
valine, leucine and isoleucine degradation
ubiquitin mediated proteolysis
tryptophan metabolism
terpenoid backbone biosynthesis
synthesis and degradation of ketone bodies
starch and sucrose metabolism
sphingolipid metabolism
rna degradation
riboflavin metabolism
protein export
propanoate metabolism
photosynthesis
phenylpropanoid biosynthesis
phagosome
peroxisome
oxidative phosphorylation
nucleotide excision repair
natural killer cell mediated cytotoxicity
mismatch repair
lysine degradation
homologous recombination
glyoxylate and dicarboxylate metabolism
glycerophospholipid metabolism
glycerolipid metabolism
fructose and mannose metabolism
fatty acid metabolism
endocytosis
carbon fixation in photosynthetic organisms
butanoate metabolism
ascorbate and aldarate metabolism
aminoacyl−trna biosynthesis
KEGG
0
5
10
15
Number of Genes
